# Modeling and Simulation of the Ion-Binding-Mediated Swelling Dynamics of Mucin-like Polyelectrolyte Gels

**DOI:** 10.3390/gels7040244

**Published:** 2021-11-30

**Authors:** Jian Du, Owen L. Lewis, James P. Keener, Aaron L. Fogelson

**Affiliations:** 1 Department of Mathematical Sciences, Florida Institute of Technology, Melbourne, FL 32901, USA; 2 Department of Mathematics and Statistics, University of New Mexico, Albuquerque, NM 87106, USA; owenlewis@unm.edu; 3 Department of Mathematics and Biomedical Engineering, University of Utah, Salt Lake City, UT 84112, USA; keener@math.utah.edu (J.P.K.); fogelson@math.utah.edu (A.L.F.)

**Keywords:** gel swelling, modeling, simulation, electrochemistry

## Abstract

Volume phase transitions in polyeletrolyte gels play important roles in many biophysical processes such as DNA packaging, nerve excitation, and cellular secretion. The swelling and deswelling of these charged polymer gels depend strongly on their ionic environment. In this paper, we present an extension to our previous two-fluid model for ion-binding-mediated gel swelling. The extended model eliminates the assumptions about the size similarity between the network and solvent particles, which makes it suitable for investigating of a large family of biologically relevant problems. The model treats the polyeletrolyte gel as a mixture of two materials, the network and the solvent. The dynamics of gel swelling is governed by the balance between the mechanical and chemical forces on each of these two materials. Simulations based on the model illustrate that the chemical forces are significantly influenced by the binding/unbinding reactions between the ions and the network, as well as the resulting distribution of charges within the gel. The dependence of the swelling rate on ionic bath concentrations is analyzed and this analysis highlights the importance of the electromigration of ions and the induced electric field in regulating gel swelling.

## 1. Introduction

Mucus is an entangled network of long-chain glycoproteins that is present in many biological systems including the respiratory and gastrointestinal tracts [1]. The mucin polymers that make up the mucus network contain numerous oligosaccharide chains which carry (depending on sulfation) negative charge groupings [2]. Therefore, mucus is often described as a polyelectrolyte gel. Like many polyelectrolyte gels, mucus can exhibit varied and dramatic swelling behaviors in response to environmental factors (pH, for example).

Measurements of the degree of swelling (relative volume change) of various polyelectrolyte gels show that samples can increase in volume by two to three orders of magnitude when immersed in solvent. Often, this swelling/hydration behavior is highly dependent on the ionic composition of the solvent. As the concentration of a monovalent salt (such as NaCl) is increased in the solvent, the equilibrium volume of the gel typically decreases in a continuous fashion [3]. It has been argued that the degree of swelling is often governed by a Donnan Equilibrium [4]. In equilibrium, there can be an electric potential difference on the order of ∼1–100 mV between the interior and exterior of the gel [3]. Thus, any quantitative model of polyelectrolyte gel swelling dynamics must incorporate a full description of the *electrochemical* potential of the network/solvent mixture. The classical description of the “energy of mixing” seen in Flory–Huggins theory is (alone) insufficient to capture the relevant phenomena [5,6].

Further complicating the issue is the fact that *divalent* salts in the solvent (for example CaCl_2_) can cause a volume phase transition in polyelectrolyte gel networks at high enough concentrations. At a critical concentration of divalent salt, the network undergoes a spontaneous collapse. This phase transition is reversible, with the gel network re-expanding after a decrease in the dissolved concentration of Ca^2+^ ions [3]. It is thought that this behavior is integral to mucus systems, as it allows for mucin networks to be produced and stored at high densities in vesicles within epithelial cells, and then to rapidly expand/swell upon exocytosis from the cells, forming a mucus layer [4]. It is believed that the collapse of the gel is due to the divalent nature of calcium and the relatively high chemical affinity of divalent ions with the polymeric network [2]. Thus, divalent ions allow for effective charge shielding of the negative groups on the network, and through association with the polymers can form transient “cross-links”, effectively altering the “mixing energy” of the solvent and network [4].

The earliest theoretical models of (not necessarily polyelectrolyte) gels attempted to describe thermodynamic equilibrium of network/solvent mixtures [5,6,7]. Later works explored non-equilibrium transient states of swelling gels [8,9,10]. More recently, much effort has been devoted to extending these models to include descriptions of the various electrical and mechanical forces which govern polyelectrolyte gel mixtures. Typically, these theoretical frameworks incorporate descriptions of the electric and chemical potentials induced by spatial gradients in the concentration of charged polymers and dissolved ions (including osmotic potential) [11,12,13,14,15]. Often the mechanical properties of the polymeric network are described as those of an elastic continuum [11,12,13,14]. However, microrheology experiments have indicated that mucus networks exhibit rheology more similar to a viscous fluid at moderate pH [16]. Some models also incorporate effects due to interfacial tension that may arise at the interface between a gel sample and the surrounding bath [13]. Many of these theoretical studies have primarily investigated equilibrium behavior of the network/solvent mixture [11,13,14]. However, some have addressed dynamic swelling behavior, and indeed a few modelling frameworks have been developed specifically to be simulated numerically [12,15]. In nearly all the extant attempts to model polyelectrolyte gel mixtures, the energy of mixing is assumed to obey the classical Flory–Huggins theory. To date, few works have attempted to generalize this mixing energy to account for ion/polymer chemistry or the particular affinity that mucin networks show for divalent cations.

The work of Sircar et al. attempted to derive, from first principles, a continuum model of a mucus-like polyelectrolyte gel that addressed these gaps in existing polyelectrolyte models. The model captures numerous classical effects, including van’t Hoff osmotic pressure, Donnan equilibrium potential, and Nernst–Planck motion of dissolved ions, as well as a Flory-like interaction parameter and a standard free energy of network phase which incorporate ionic binding chemistry (and distinguish between chemical interactions with monovalent vs. divalent cations). Sircar et al. outlined the derivation of the model and presented an equilibrium analysis of it [17]. Later work has investigated the dynamic swelling behavior of the model by investigating linear stability of equilibrium configurations and the near-equilibrium dynamics [18] or by performing (one-dimensional) numerical simulations away from equilibrium in a drag dominated parameter regime (i.e., very low Darcy permeability of the gel) [19]. Only recently have numerical techniques been developed to simulate the model in higher dimensions with reasonably accuracy and efficiency [20]. However, an assumption implicit in the derivation in [17], is that the monomeric units which make up the polymeric network are of *approximately* the same volume as solvent particles (water), and can therefore be placed on the same lattice for the purposes of statistical-mechanical calculations. This assumption is unlikely to be valid for mucus networks. Furthermore, the model of Sircar et al. is analyzed in terms of the electrochemical potentials acting on the various particle species which make up the polyelectrolyte mixture (network, solvent, dissolved ions). This simplifies the presentation of equilibrium calculations. However, the dynamic rearrangement of the network is governed by force densities (i.e., potential gradients), and expressing the model in terms of potentials obfuscates various subtle effects which affect dynamic swelling behaviors.

The goal of this work is therefore threefold: (1) to re-derive (from first principles) the equations of motion for a model of a mucus-like polyelectrolyte gel based on the work of Sircar et al. without the assumption that network and solvent particles are of similar size, (2) to formulate the model in terms of a balance of force densities acting on the network and solvent phases, for clarity, and (3) to simulate and analyze a set of numerical experiments in which a dense sample of mucus, with a high concentration of calcium, is placed into a bath, in which monovalent sodium ions far outnumbered divalent calcium ones, and is allowed to swell dynamically. In Section 2 we give a detailed description of our model assumptions and a derivation of the model equations. The motion of the network and solvent phases of the gel may be attributed to six force densities. Three of these force densities are electrochemical in nature; the remaining three are mechanical in nature. In Section 3, we briefly outline the numerical scheme used to simulate the model in two spatial dimensions (for a detailed description of the numerics, please see [20]). In Section 4, we describe our experiments which simulate a sample of dense mucus allowed to swell in a bath of known ionic composition. We show that samples placed into baths of differing ionic strength swell at differing rates. This variation in the swelling rate is explained in terms of the various forces acting on the network. Broadly speaking, we can understand the dynamic movement of the network/solvent phases as being driven by the three electrochemical force densities, while the mechanical forces arise in response to the motion of the two phases.

## 2. Mathematical Model

Following [17], we develop a model of polyelectrolyte gels built upon the “two-phase” model framework. We consider a polymer gel which is a multi-component material made up of polymers, water (solvent), and several distinct types of ions. The polymer is made up of “monomeric units”, denoted M, each of which carries a single negative charge and is capable of forming a single bond with a cation dissolved in the solvent. Ionic species may be either positively or negatively charged. Cations may be mono- or divalent, however, for simplicity we assume all anionic species are monovalent. In this text we refer to monovalent cations as sodium, divalent cations as calcium, and anions as chloride, but the modeling framework is by no means specific to these ionic species. Chloride is assumed to be unable to participate in any binding chemistry with the polymeric network, but must be present in the solvent as a counterion. The relevant chemical reactions are

(1) $$M^{-}+{\mathrm{Na}}^{+}⇌\mathrm{MNa},\;M^{-}+{\mathrm{Ca}}^{2+}⇌M{\mathrm{Ca}}^{+},\;M^{-}+M{\mathrm{Ca}}^{+}⇌M_{2}Ca.$$

Here, M^−^ denotes a monomeric unit not bound to an ion, MNa and MCa^+^ denote a monomeric unit bound to a sodium and calcium ion, respectively, and M_2_Ca denotes two monomeric units cross-linked by a calcium ion. These species are all assumed to be part of the polymeric network and move with the same velocity as the network. The species M_2_Ca plays an important role in determining the dynamics of the polymer network.

An assumption underlying the model developed in [17] is that all particles within the gel are of *roughly* the same size. Thus, for statistical arguments involving lattice configurations, all particles could be placed on the *same* lattice. In the case that the characteristic volume of a monomeric unit M is much larger than that of a water molecule or individual ion, this assumption may be inappropriate. To remedy this issue, we define an auxiliary “particle” which we refer to as a “solvent aggregate”. This is defined to be a contiguous collection of water molecules and ions which occupy a volume equal to that of a single monomeric unit. We denote the volume of monomeric units M and solvent aggregates as $\nu_{n}$. The volume of an individual solvent particle or ion is denoted $\nu_{s}$ and is taken to be equal to the volume of a water molecule.

The experiments of [21] suggest that hydrated gastric mucus may bind cations in concentrations on the order of 100 mM. If we assume that mucus is between 2–10% polymeric network by volume, this implies a concentration of “monomeric units” on the order of 1–5 M in pure mucus, which in turn implies that $\nu_{n}\approx 10\nu_{s}$–50$\nu_{s}$. However, other experiments conducted on *salivary* mucus suggest that mucus networks exhibit binding sites in significantly lower densities, which in turn implies $\nu_{n}>>50\nu_{s}$ [22]. Based on these estimates, we assume $\gamma =\nu_{s}/\nu_{n}<<1$. We denote the (molar) concentration of monomeric units in pure network by $m^{\mathrm{tot}}$ and the (molar) concentration of water molecules in pure water by $s^{\mathrm{tot}}$ (≈55.5 M) and we note that $\nu_{n}m^{\mathrm{tot}}=\nu_{s}s^{\mathrm{tot}}$. The actual concentration of particles in a solvent aggregate may deviate very slightly from $s^{\mathrm{tot}}$ because of binding/unbinding of ions to the network. For ions with concentrations much lower than $s^{\mathrm{tot}}$ (dilute), the number of molecules in a solvent aggregate will remain very close to $\nu_{n}/\nu_{s}$. We restrict our attention to such dilute ion solutions, and below make the approximation that on average there are always $\nu_{n}/\nu_{s}$ molecules in a solvent aggregate, or equivalently a concentration of $s^{\mathrm{tot}}$ in solvent aggregates. Note that we do not assume that the polymer is dilute.

### 2.1. Equations of Motion

By assumption, the polymer network and the solvent aggregates are the two phases which partition the volume of the mixture. If $N_{n}$ and $N_{s}$ are the number of monomeric units and solvent aggregates in a volume, then we may define their volume fractions $\theta_{i}$ and number fractions $\phi_{i}$, respectively, by

(2) $$\theta_{n}=\phi_{n}=\frac{N_{n}}{N_{s}+N_{n}},$$

(3) $$\theta_{s}=\phi_{s}=\frac{N_{s}}{N_{s}+N_{n}}.$$

For brevity, we refer to these two volume occupying phases as the “network” and “solvent”. When it is necessary for clarity below, we are explicit in referring to individual solvent particles/molecules (which make up a large fraction, but not all of the solvent phase). We assume that each phase is transported with its own velocity, and thus conservation of mass yields the evolution equations

(4) $$\frac{\partial \theta_{n}}{\partial t}+\nabla \cdot \left(u_{n}\theta_{n}\right)=0,$$

(5) $$\frac{\partial \theta_{s}}{\partial t}+\nabla \cdot \left(u_{s}\theta_{s}\right)=0,$$

where $u_{n}$ and $u_{s}$ are the velocity of the network and solvent phases, respectively. As $\theta_{n}+\theta_{s}=1$, we have the “volume averaged” incompressibility constraint

(6) $$\nabla \cdot \left(u_{n}\theta_{n}+u_{s}\theta_{s}\right)=0.$$

The minimum rate of work principle (sometimes referred to as the Helmholtz minimum energy dissipation rate principle) may be used to derive a set of force balance equations which govern the velocity fields of the network, solvent, and individual particles which make up the solvent aggregates.

(7) $$\nabla \cdot \left(\theta_{n}{\underline{\sigma }}_{n}\left(u_{n}\right)\right)-\frac{\xi }{\nu_{n}}\theta_{n}\theta_{s}\left(u_{n}-u_{s}\right)-\frac{\theta_{n}}{\nu_{n}}\nabla \mu_{n}-\theta_{n}\nabla p=0,$$

(8) $$\nabla \cdot \left(\theta_{s}{\underline{\sigma }}_{s}\left(u_{s}\right)\right)-\frac{\xi }{\nu_{n}}\theta_{n}\theta_{s}\left(u_{s}-u_{n}\right)-\frac{\theta_{s}}{\nu_{n}}\nabla \mu_{s}-\theta_{s}\nabla p+\frac{\theta_{s}}{\nu_{s}}\sum_{j}{\hat{\xi }}_{j}{\hat{\phi }}_{j}\left(u_{j}-u_{s}\right)=0.$$

(9) $${\hat{\xi }}_{j}{\hat{\phi }}_{j}\left(u_{s}-u_{j}\right)-{\hat{\phi }}_{j}\nabla \mu_{j}=0,\mathrm{for}j=1,\ldots ,N_{\mathrm{ions}}\mathrm{and}H_{2}O,$$

where $N_{\mathrm{ions}}$ is the number of distinct species of ions dissolved in the solvent and $j=H_{2}O$ designates water molecules. In Equations (7) and (8), ${\underline{\sigma }}_{i}$ is the viscous stress tensor of phase i (i=s,n), ξ is the coefficient for the drag arising from relative motion of network and solvent, ${\hat{\xi }}_{j}$ is the coefficient for the drag between the jth molecular species in the solvent and the solvent as a whole, and *p* is the hydrodynamic pressure. The quantities ${\hat{\phi }}_{j}$ denote the number fractions of ions and individual solvent particles which make up the solvent aggregates. If the numbers of individual solvent particles and ions of type j contained in *all* the solvent aggregates within a given volume are denoted $N_{H_{2}O}$ and $N_{j}$, respectively, then

(10) $${\hat{\phi }}_{H_{2}O}=\frac{N_{H_{2}O}}{N_{H_{2}O}+\sum_{k\neq H_{2}O}N_{k}},\;{\hat{\phi }}_{j}=\frac{N_{j}}{N_{H_{2}O}+\sum_{k\neq H_{2}O}N_{k}},$$

where the sums are over all of the dissolved ion species. We note here that each molecular species in the solvent aggregates has its distinct velocity field, which is governed by its force balance Equation (9). In Section 2.3, we show that these equations may be used to eliminate the individual particle velocities. The net effect is that the solvent aggregates “feel” the forces which act on their constituent particles. See [17] for more details.

The network phase n is acted upon by the electric potential Ψ (as it may carry charge), entropy, and short-range interactions due to the arrangement of monomeric units and solvent aggregates. Solvent aggregates experience potentials due to the same short-range interactions. The particles which make up the solvent aggregates are acted upon by entropy and, in the case of ions, by the electric potential. We do not account for other interactions between water molecules and ions because the ions are assumed to be dilute. Hence, the chemical potentials for network, solvent aggregates, water, and ions have the form:

(11) $$\mu_{n}=\mu_{n}^{E}+\mu_{n}^{S}+\mu_{n}^{I},$$

(12) $$\mu_{s}=\mu_{s}^{I},$$

(13) $$\mu_{H_{2}O}=\mu_{H_{2}O}^{S},$$

(14) $$\mu_{j}=\mu_{j}^{E}+\mu_{j}^{S},\;j\neq H_{2}O.$$

where *S*, *E*, and *I* denote entropic, electrical, and short-range interaction potentials.

### 2.2. Potentials Acting on Particles

In this section we derive the electrochemical potentials that act on the various species. In the interest of brevity, we refer the reader to [17] for details when necessary. In Section 2.3, we calculate the force densities which appear in Equations (7) and (8) and show that the equations of motion may be simplified.

#### 2.2.1. Entropy

If the mixture contains $N_{s}$ solvent aggregates and $N_{n}$ monomeric units, then the number of possible arrangements of these particles on a lattice is given by

(15) $$\Omega_{\mathrm{meso}}=\frac{(N_{n}+N_{s})!}{N_{n}!N_{s}!}.$$

The number of individual solvent particles contained in all of the solvent aggregates is denoted $N_{H_{2}O}$ and the number of ions of type j is denoted $N_{j}$. The number of ways that these particles can be arranged within all $N_{s}$ solvent aggregates is given by

(16) $$\Omega_{\mathrm{micro}}=\frac{\left(N_{H_{2}O}+\sum_{j\neq H_{2}O}N_{j}\right)!}{N_{H_{2}O}!\prod_{j\neq H_{2}O}N_{j}!}$$

Equations (15) and (16) imply that the total number of ways to arrange particles in the system is given by

(17) $$\Omega =\frac{(N_{n}+N_{s})!}{N_{n}!N_{s}!}\frac{\left(N_{H_{2}O}+\sum_{j\neq H_{2}O}N_{j}\right)!}{N_{H_{2}O}!\prod_{j\neq H_{2}O}N_{j}!},$$

and so the entropy of the mixture is

(18) $$S=k_{B}\ln\left(\Omega \right)=k_{B}\left(\ln\left(\frac{(N_{n}+N_{s})!}{N_{n}!N_{s}!}\right)+\ln\left(\frac{\left(N_{H_{2}O}+\sum_{j\neq H_{2}O}N_{j}\right)!}{N_{H_{2}O}!\prod_{j\neq H_{2}O}N_{j}!}\right)\right).$$

Here $k_{B}$ is the Boltzmann constant. Using Stirling’s formula (ln(N!)≈Nln(N)−N), this may be simplified to

(19) $$\begin{array}{c} \frac{S}{k_{B}}=-N_{n}\ln\left(\frac{N_{n}}{N_{n}+N_{s}}\right)-N_{s}\ln\left(\frac{N_{s}}{N_{n}+N_{s}}\right)\\ \;\;-N_{H_{2}O}\ln\left(\frac{N_{H_{2}O}}{N_{H_{2}O}+\sum_{k\neq H_{2}O}N_{k}}\right)-\sum_{j\neq H_{2}O}N_{j}\ln\left(\frac{N_{j}}{N_{H_{2}O}+\sum_{k\neq H_{2}O}N_{k}}\right). \end{array}$$

This expression assumes that the monomeric units can be arranged arbitrarily. For polymers which are composed of chains of $N_{\mathrm{chain}}$ monomeric units, standard arguments result in the factor of $N_{n}$ in front of the leading term being replaced by $N_{n}/N_{\mathrm{chain}}$ [23]. The entropy function then becomes

(20) $$\begin{array}{c} \frac{S}{k_{B}}=-\frac{N_{n}}{N_{\mathrm{chain}}}\ln\left(\frac{N_{n}}{N_{n}+N_{s}}\right)-N_{s}\ln\left(\frac{N_{s}}{N_{n}+N_{s}}\right)\\ \;\;-N_{H_{2}O}\ln\left(\frac{N_{H_{2}O}}{N_{H_{2}O}+\sum_{k\neq H_{2}O}N_{j}}\right)-\sum_{j\neq H_{2}O}N_{j}\ln\left(\frac{N_{j}}{N_{H_{2}O}+\sum_{k\neq H_{2}O}N_{k}}\right). \end{array}$$

This is the total entropy of the mixture. Note that by the definition of $\nu_{s}$ and $\nu_{n}$, and the relationship $\nu_{n}m^{\mathrm{tot}}=\nu_{s}s^{\mathrm{tot}}$,

(21) $$N_{s}=\gamma \left(N_{H_{2}O}+\sum_{j\neq H_{2}O}N_{j}\right),\;\gamma =\frac{\nu_{s}}{\nu_{n}}.$$

Letting *T* be the temperature, the potential due to entropy for each particle species $k=n,j,\mathrm{and}H_{2}O$ is given by $\mu_{k}^{S}=-T\frac{\partial S}{\partial N_{k}}$. The entropy of the network/polymer phase is the simplest. Using the definition of particle fraction (see Equations (2) and (3)), we have

(22) $$\begin{array}{cc} \frac{\mu_{n}^{S}}{k_{B}T} & =\frac{1}{N_{\mathrm{chain}}}\ln\left(\frac{N_{n}}{N_{n}+N_{s}}\right)-\left(1-\frac{1}{N_{\mathrm{chain}}}\right)\frac{N_{s}}{N_{n}+N_{s}}\\ \; & =\frac{1}{N_{\mathrm{chain}}}\ln\left(\phi_{n}\right)-\left(1-\frac{1}{N_{\mathrm{chain}}}\right)\phi_{s}. \end{array}$$

Using Equation (21), from which we see that $\frac{\partial N_{s}}{\partial N_{H_{2}O}}=\frac{\partial N_{s}}{\partial N_{j}}=\gamma$, a similar calculation gives the entropic potential for each type of solvent particle:

(23) $$\begin{array}{cc} \frac{\mu_{H_{2}O}^{S}}{k_{B}T} & =\gamma \left(\ln\left(\frac{N_{s}}{N_{n}+N_{s}}\right)+\left(1-\frac{1}{N_{\mathrm{chain}}}\right)\frac{N_{n}}{N_{n}+N_{s}}\right)+\ln\left(\frac{N_{H_{2}O}}{N_{H_{2}O}+\sum_{j\neq H_{2}O}N_{j}}\right)\\ \; & =\gamma \left(\ln\left(\phi_{s}\right)+\left(1-\frac{1}{N_{\mathrm{chain}}}\right)\phi_{n}\right)+\ln\left({\hat{\phi }}_{H_{2}O}\right), \end{array}$$

and

(24) $$\begin{array}{cc} \frac{\mu_{j}^{S}}{k_{B}T} & =\gamma \left(\ln\left(\frac{N_{s}}{N_{n}+N_{s}}\right)+\left(1-\frac{1}{N_{\mathrm{chain}}}\right)\frac{N_{n}}{N_{n}+N_{s}}\right)+\ln\left(\frac{N_{j}}{N_{H_{2}O}+\sum_{j}N_{j}}\right)\\ \; & =\gamma \left(\ln\left(\phi_{s}\right)+\left(1-\frac{1}{N_{\mathrm{chain}}}\right)\phi_{n}\right)+\ln\left({\hat{\phi }}_{j}\right). \end{array}$$

Here, $\phi_{s}$ is the particle fraction of solvent aggregates in the two phases, while ${\hat{\phi }}_{H_{2}O}$ and ${\hat{\phi }}_{j}$ are the particle fraction of solvent particles and of ions of type j within solvent aggregates, respectively.

#### 2.2.2. Electrostatic Potential

The network contains variable concentrations of negatively charged M^−^ units and positively charged MCa^+^ units, as well as concentrations of two uncharged units, MNa and M_2_Ca. We define the variables $B_{\mathrm{MNa}},$ $B_{\mathrm{MCa}},$ and $B_{M_{2}\mathrm{Ca}}$ to be the molar concentration (per *total volume*) of MNa, MCa^+^, and M_2_Ca, respectively. Notice that the values of $B_{\mathrm{MNa}},$ $B_{\mathrm{MCa}},$ and $B_{M_{2}\mathrm{Ca}}$ also represent the concentrations of bound sodium, calcium bound only to one monomeric unit (referred to as singly-bound calcium), and calcium bound to two monomeric units (referred to as doubly-bound calcium), respectively. The concentration of negatively charged M^−^ units is given by $m=m^{\mathrm{tot}}\theta_{n}-B_{\mathrm{MNa}}-B_{\mathrm{MCa}}-2B_{M_{2}\mathrm{Ca}}$, where, recall, $m^{\mathrm{tot}}$ is the (molar) concentration of monomeric units (and hence negative charge) in a sample of pure mucin. The net concentration of positive charge on the network is $B_{\mathrm{MNa}}+2B_{\mathrm{MCa}}+2B_{M_{2}\mathrm{Ca}}-m^{\mathrm{tot}}\theta_{n}$, and the average number of (positive) charges per monomeric unit is $z_{m}$ defined by

(25) $$z_{m}=\frac{B_{\mathrm{MNa}}+2B_{\mathrm{MCa}}+2B_{M_{2}\mathrm{Ca}}-m^{\mathrm{tot}}\theta_{n}}{m^{\mathrm{tot}}\theta_{n}}.$$

The electrostatic force on the network, as described in more detail below, is proportional to $z_{m}$, the number density of monomeric units, $\theta_{n}/\nu_{n}$, and the gradient of the electrostatic potential Ψ. Similarly, the electrostatic force acting on ions of type j with valence $z_{j}$ is proportional to $z_{j}$, the number density of that type of ion, and the gradient of Ψ.

#### 2.2.3. Short-Range Interactions

Following the approach of [17], we envision monomeric units and solvent aggregates within a volume arranged on a lattice with coordination number *z*. The fraction of monomeric units which are cross-linked via calcium binding (i.e., the fraction of M which are in state M_2_Ca) is denoted α, and is given by the expression

$$\alpha =\frac{2B_{M_{2}\mathrm{Ca}}}{m+B_{\mathrm{MNa}}+B_{\mathrm{MCa}}+2B_{M_{2}\mathrm{Ca}}}=\frac{2B_{M_{2}\mathrm{Ca}}}{m^{\mathrm{tot}}\theta_{n}}.$$

We denote the pair-wise interaction energies between an adjacent pair of cross-linked monomers, an adjacent pair of uncross-linked monomers, two adjacent monomers in the same polymer chain, an adjacent monomer/solvent aggregate pair, and a pair of adjacent solvent aggregates as $k_{B}T\epsilon_{\mathrm{xx}}$, $k_{B}T\epsilon_{\mathrm{uu}}$, $k_{B}T\epsilon_{\mathrm{pp}}$, $k_{B}T\epsilon_{\mathrm{us}}$, and $k_{B}T\epsilon_{\mathrm{ss}}$, respectively. Using a standard mean-field counting argument (see [17]), the total *per-particle* interaction energy is given by

(26) $$U^{I}=k_{B}T\left(\frac{\chi (\alpha )}{2}\phi_{n}\phi_{s}+\mu_{s}^{0}\phi_{s}+\mu_{n}^{0}\phi_{n}\right),$$

where

(27) $$\chi \left(\alpha \right)=z\left(2\epsilon_{\mathrm{us}}-\epsilon_{\mathrm{uu}}-\epsilon_{\mathrm{ss}}\right)-2\left(1-\frac{1}{N_{\mathrm{chain}}}\right)\left(\epsilon_{\mathrm{us}}-\epsilon_{\mathrm{uu}}\right)-\left(\epsilon_{\mathrm{us}}-\epsilon_{\mathrm{uu}}\right)\alpha ,$$

(28) $$\mu_{n}^{0}\left(\alpha \right)=\epsilon_{\mathrm{uu}}\frac{z}{2}+\left(\epsilon_{\mathrm{pp}}-\epsilon_{\mathrm{uu}}\right)\left(1-\frac{1}{N_{\mathrm{chain}}}\right)+\frac{\alpha }{2}\left(\epsilon_{\mathrm{xx}}-\epsilon_{\mathrm{uu}}\right),$$

(29) $$\mu_{s}^{0}=\epsilon_{\mathrm{ss}}\frac{z}{2},$$

and $\phi_{n}$ and $\phi_{s}$ are the particle fractions defined in Equations (2) and (3). We refer to χ(α) as the “interaction parameter”. The dependence of χ(α) and the polymer standard free energy $\mu_{n}^{0}\left(\alpha \right)$ on α determine the relationship between the free energy of the gel mixture and the fraction of monomers cross-linked by divalent calcium. The total short-range interaction energy of the mixture is given by

(30) $$E^{I}=\left(N_{s}+N_{n}\right)U^{I},$$

and therefore the short-range interaction potentials acting on the two phases are calculated as

(31) $$\mu_{n}^{I}=\frac{\partial E^{I}}{\partial N_{n}}=k_{B}T\left(\frac{\chi (\alpha )}{2}\phi_{s}^{2}+\mu_{n}^{0}\left(\alpha \right)\right),$$

(32) $$\mu_{s}^{I}=\frac{\partial E^{I}}{\partial N_{s}}=k_{B}T\left(\frac{\chi (\alpha )}{2}\phi_{n}^{2}+\mu_{s}^{0}\right).$$

We note that in the case α=0, Equation (30) reduces to the interaction energy found in the standard Flory–Huggins theory [23]. However, α often varies in space and time (discussed below), and then the interaction parameter and the standard free energy of the network phase also vary in space and time. In particular, the standard free energy $\mu_{n}^{0}\left(\alpha \right)$ may give rise to forces on the network. This is not a feature of many classical theories [17].

### 2.3. Forces Acting on the System

Before discussing the dynamics of the ion species, we first recast the equations for the network and solvent dynamics in terms of the force densities acting on the system. This is useful for interpreting later results and also facilitates simplifying the model equations.

First, we note that Equation (9) can be used in Equation (8) to eliminate the drag force density resulting from relative motion between solvent aggregates as a whole and the individual molecular species in the solvent aggregates. Doing so yields the force balance equations

(33) $$\nabla \cdot \left(\theta_{n}{\underline{\sigma }}_{n}\left(u_{n}\right)\right)-\frac{\xi }{\nu_{n}}\theta_{n}\theta_{s}\left(u_{n}-u_{s}\right)-\theta_{n}\nabla p-\frac{\theta_{n}}{\nu_{n}}\nabla \mu_{n}^{I}-\frac{\theta_{n}}{\nu_{n}}\nabla \mu_{n}^{S}-\frac{\theta_{n}}{\nu_{n}}\nabla \mu_{n}^{E}=0,$$

(34) $$\nabla \cdot \left(\theta_{s}{\underline{\sigma }}_{s}\left(u_{s}\right)\right)-\frac{\xi }{\nu_{n}}\theta_{n}\theta_{s}\left(u_{s}-u_{n}\right)-\theta_{s}\nabla p-\frac{\theta_{s}}{\nu_{n}}\nabla \mu_{s}^{I}-\frac{\theta_{s}}{\nu_{s}}\sum_{j}{\hat{\phi }}_{j}\left(\nabla \mu_{j}^{S}+\nabla \mu_{j}^{E}\right)=0.$$

for the network and solvent phases, respectively. The sum in Equation (34), is over $j=H_{2}O$ and $j=1,\ldots ,N_{\mathrm{ions}}$. For $j=H_{2}O$, $\mu_{j}^{E}=0$.

#### 2.3.1. Entropic Forces

Evaluating the force densities which arise from the entropic potentials given in Equations (22)–(24), we have

(35) $$\begin{array}{cc} f_{n}^{S} & =-\frac{\theta_{n}}{\nu_{n}}\nabla \mu_{n}^{S}\\ \; & =-\frac{k_{B}T}{\nu_{n}}\left(\frac{1}{N_{\mathrm{chain}}}+\left(1-\frac{1}{N_{\mathrm{chain}}}\right)\theta_{n}\right)\nabla \theta_{n}, \end{array}$$

(36) $$\begin{array}{cc} f_{H_{2}O}^{S} & =-\frac{\theta_{s}{\hat{\phi }}_{H_{2}O}}{\nu s}\nabla \mu_{H_{2}O}^{S}\\ \; & =-\frac{k_{B}T}{\nu_{s}}\left[\gamma {\hat{\phi }}_{H_{2}O}\left(\left(\frac{1}{N_{\mathrm{chain}}}-1\right)\theta_{s}\nabla \theta_{s}+\nabla \theta_{s}\right)+\theta_{s}\nabla {\hat{\phi }}_{H_{2}O}\right], \end{array}$$

(37) $$\begin{array}{cc} f_{j}^{S} & =-\frac{\theta_{s}{\hat{\phi }}_{j}}{\nu_{s}}\nabla \mu_{j}^{S}\\ \; & =-\frac{k_{B}T}{\nu_{s}}\left[\gamma {\hat{\phi }}_{j}\left(\left(\frac{1}{N_{\mathrm{chain}}}-1\right)\theta_{s}\nabla \theta_{s}+\nabla \theta_{s}\right)+\theta_{s}\nabla {\hat{\phi }}_{j}\right]. \end{array}$$

From Equation (34), the total force density (due to entropy) acting on the solvent phase is given as the sum of the force densities $f_{H_{2}O}$ and $f_{j},\;\;j=1,\ldots ,N_{\mathrm{ions}}$. Using Equations (36) and (37) to compute this sum and, using the fact that ${\hat{\phi }}_{H_{2}O}+\sum_{j\neq H_{2}O}{\hat{\phi }}_{j}=1$, we can express the total entropic force density on the solvent phase as

(38) $$\begin{array}{cc} f_{s}^{S} & =f_{H_{2}O}^{S}+\sum_{j\neq H_{2}O}f_{j}^{S}\\ \; & =-\frac{k_{B}T}{\nu_{s}}\left[\gamma \left(\left(\frac{1}{N_{\mathrm{chain}}}-1\right)\theta_{s}\nabla \theta_{s}+\nabla \theta_{s}\right)\right]\\ \; & =-\frac{k_{B}T}{\nu_{n}}\left(\left(\frac{1}{N_{\mathrm{chain}}}-1\right)\theta_{s}\nabla \theta_{s}+\nabla \theta_{s}\right). \end{array}$$

Equation (38) implies that entropy due to rearrangements of individual solvent/ion particles within the solvent aggregates does not produce any net forces on the solvent phase. Only rearrangements of network particles and solvent aggregates lead to forces on these two phases and contribute to driving their dynamics. Further, it follows from Equations (35) and (38), and the identity $\theta_{n}+\theta_{s}=1$, that $f_{s}^{S}+f_{n}^{S}=0$ everywhere. This simplifies calculations (and numerical simulations) greatly. Finally, we note that Equations (35) and (38) recapitulate standard expressions for forces due to entropy in a two-species polymer solution [23].

#### 2.3.2. Electric Force Densities

From Equation (34) we can express the force density that the solvent phase experiences due to the electric potential gradient acting on dissolved ions as

(39) $$f_{s}^{E}=-\frac{\theta_{s}}{\nu_{s}}\sum_{j}{\hat{\phi }}_{j}\nabla \mu_{j}^{E}=-\frac{\theta_{s}}{\nu_{s}}\sum_{j}{\hat{\phi }}_{j}z_{j}k_{B}T\nabla \Psi ,$$

where the sum is over $j=1,\ldots ,N_{\mathrm{ions}}$ since $\nabla \mu_{H_{2}O}^{E}\equiv 0$. Using the definition of the number fraction ${\hat{\phi }}_{j}$ from Equation (10) we have

(40) $$f_{s}^{E}=-\frac{\theta_{s}}{\nu_{s}}\frac{N_{\mathrm{Na}}+2N_{\mathrm{Ca}}-N_{\mathrm{Cl}}}{N_{H_{2}O}+N_{\mathrm{Na}}+N_{\mathrm{Ca}}+N_{\mathrm{Cl}}}k_{B}T\nabla \Psi .$$

Dividing the numerator and denominator by Avogadro’s number times the total volume of the solvent aggregates ($N_{A}N_{s}\nu_{n}$), Equation (40) becomes

(41) $$\begin{array}{ccc} f_{s}^{E} & = & -\frac{\theta_{s}}{\nu_{s}}\frac{C_{\mathrm{Na}}+2C_{\mathrm{Ca}}-C_{\mathrm{Cl}}}{C_{H_{2}O}+C_{\mathrm{Na}}+C_{\mathrm{Ca}}+C_{\mathrm{Cl}}}k_{B}T\nabla \Psi \\ & = & -\frac{\theta_{s}}{\nu_{s}}\frac{C_{\mathrm{Na}}+2C_{\mathrm{Ca}}-C_{\mathrm{Cl}}}{s^{\mathrm{tot}}}k_{B}T\nabla \Psi , \end{array}$$

where the variables $C_{j}$ denote concentrations of the respective ions and $C_{H_{2}O}$ is the concentration of water molecules. These concentrations are calculated in a volume where no polymer/network exists, and thus they correspond to “moles of ion per unit of solvent volume”. The equations of motion for $C_{j}$ are discussed in more detail below. Since the overall material is electrically neutral,

(42) $$B_{\mathrm{MNa}}+2B_{\mathrm{MCa}}+2B_{M_{2}\mathrm{Ca}}-m^{\mathrm{tot}}\theta_{n}+\theta_{s}\left(C_{\mathrm{Na}}+2C_{\mathrm{Ca}}-C_{\mathrm{Cl}}\right)=0,$$

it follows that the force densities due to electric potential gradients on the two materials must be equal and opposite. We can verify that this is true as follows. The electric force density on the network is

(43) $$f_{n}^{E}=-\frac{\theta_{n}}{\nu_{n}}\nabla \mu_{n}^{E}=-\frac{\theta_{n}}{\nu_{n}}\frac{B_{\mathrm{MNa}}+2B_{\mathrm{MCa}}+2B_{M_{2}\mathrm{Ca}}-m^{\mathrm{tot}}\theta_{n}}{m^{\mathrm{tot}}\theta_{n}}k_{B}T\nabla \Psi .$$

and, from Equation (41), the electric force density acting on the solvent aggregates is

(44) $$f_{s}^{E}=-\frac{\theta_{s}}{\nu_{s}}\nabla \mu_{n}^{E}=-\frac{\theta_{s}}{\nu_{s}}\frac{C_{\mathrm{Na}}+2C_{\mathrm{Ca}}-C_{\mathrm{Cl}}}{s^{\mathrm{tot}}}k_{B}T\nabla \Psi .$$

Hence,

(45) $$f_{n}^{E}+f_{s}^{E}=-\left[\frac{\theta_{n}}{\nu_{n}}\frac{B_{\mathrm{MNa}}+2B_{\mathrm{MCa}}+2B_{M_{2}\mathrm{Ca}}-m^{\mathrm{tot}}\theta_{n}}{m^{\mathrm{tot}}\theta_{n}}+\frac{\theta_{s}}{\nu_{s}}\frac{C_{\mathrm{Na}}+2C_{\mathrm{Ca}}-C_{\mathrm{Cl}}}{s^{\mathrm{tot}}}\right]k_{B}T\nabla \Psi .$$

Since $\nu_{n}m^{\mathrm{tot}}=\nu_{s}s^{\mathrm{tot}}$, this simplifies to

(46) $$f_{n}^{E}+f_{s}^{E}=-\frac{1}{m^{\mathrm{tot}}\nu_{n}}\left(B_{\mathrm{MNa}}+2B_{\mathrm{MCa}}+2B_{M_{2}\mathrm{Ca}}-m^{\mathrm{tot}}\theta_{n}+\theta_{s}\left(C_{\mathrm{Na}}+2C_{\mathrm{Ca}}-C_{\mathrm{Cl}}\right)\right)\nabla \Psi ,$$

which vanishes because of the electroneutrality condition Equation (42). Note that Equations (43) and (44) can be written as $f_{n}^{E}=-\frac{\theta_{n}}{\nu_{n}}z_{m}k_{B}T\nabla \Psi$ and $f_{s}^{E}=-\frac{\theta_{s}}{\nu_{s}}z_{s}k_{B}T\nabla \Psi$, respectively, where $z_{m}$, given in Equation (25), is the average charge per monomeric unit of network and $z_{s}=\frac{C_{\mathrm{Na}}+2C_{\mathrm{Ca}}-C_{\mathrm{Cl}}}{s^{\mathrm{tot}}}$ is the average charge per particle in a solvent aggregate.

#### 2.3.3. Short-Range Interactions

For completeness, we note here that the forces exerted on the respective network and solvent phases due to short range interactions are given by the gradient of the corresponding potential

(47) $$f_{n}^{I}=-\frac{\theta_{n}}{\nu_{n}}\nabla \mu_{n}^{I}=-k_{B}T\frac{\theta_{n}}{\nu_{n}}\nabla \left(\frac{\chi (\alpha )}{2}\theta_{s}^{2}+\mu_{n}^{0}\left(\alpha \right)\right),$$

(48) $$f_{s}^{I}=-\frac{\theta_{s}}{\nu_{n}}\nabla \mu_{s}^{I}=-k_{B}T\frac{\theta_{s}}{\nu_{n}}\nabla \left(\frac{\chi (\alpha )}{2}\theta_{n}^{2}+\mu_{s}^{0}\right).$$

Based on all of the chemical forces defined above, we rewrite the force balance equations for the network and solvent as:

(49) $$\nabla \cdot \left(\theta_{n}{\underline{\sigma }}_{n}\left(u_{n}\right)\right)-\frac{\xi }{\nu_{n}}\theta_{n}\theta_{s}\left(u_{n}-u_{s}\right)-\theta_{n}\nabla p+f_{n}^{E}+f_{n}^{I}+f_{n}^{S}=0,$$

(50) $$\nabla \cdot \left(\theta_{s}{\underline{\sigma }}_{s}\left(u_{s}\right)\right)-\frac{\xi }{\nu_{n}}\theta_{n}\theta_{s}\left(u_{s}-u_{n}\right)-\theta_{s}\nabla p+f_{s}^{E}+f_{s}^{I}+f_{s}^{S}=0,$$

where the various forces are defined by Equations (35), (38), (41), (43), (47), and (48).

### 2.4. Chemical Evolution

In this section, we present the equations of motion for the bound and dissolved ionic species. As previously stated, the concentration (per total volume) of bound sodium, singly bound calcium, and doubly bound calcium are denoted $B_{\mathrm{MNa}}$, $B_{\mathrm{MCa}}$, and $B_{M_{2}\mathrm{Ca}}$, respectively. We denote the concentration of *unbound* monomeric particles *M* as *m*. Then, conservation of monomeric particles implies

(51) $$m=m^{\mathrm{tot}}\theta_{n}-\left(B_{\mathrm{MNa}}+B_{\mathrm{MCa}}+2B_{M_{2}\mathrm{Ca}}\right).$$

The concentrations of dissolved sodium, dissolved calcium, and dissolved chloride (measured per unit of *solvent* volume) are denoted $C_{\mathrm{Na}}$, $C_{\mathrm{Ca}}$, and $C_{\mathrm{Cl}}$, respectively.

We may use Equation (9) to solve for each of the ion velocities and then use the result to obtain the expression

(52) $$J_{j}=C_{j}u_{j}=C_{j}\left(u_{s}-\frac{1}{{\hat{\xi }}_{j}}\nabla \mu_{j}\right).$$

for the flux of dissolved ion j. The total potential acting on the *j*th dissolved ionic species is given by

(53) $$\frac{\mu_{j}}{k_{B}T}=z_{j}\Psi +\gamma \left(\ln\left(\phi_{s}\right)+\left(1-\frac{1}{N_{\mathrm{chain}}}\right)\phi_{n}\right)+\ln\left({\hat{\phi }}_{j}\right).$$

Since $\gamma =\nu_{s}/\nu_{n}<<1$, we can express the ion flux, to leading order, as

(54) $$J_{j}=u_{s}C_{j}-D_{j}\left(\nabla C_{j}+z_{j}C_{j}\nabla \Psi \right),$$

where $D_{j}=k_{B}T/{\hat{\xi }}_{j}$. Equation (54) is equivalent to a classical Nernst–Planck flux. Conservation of mass for ion j may be expressed as

(55) $$\frac{\partial }{\partial t}\left(\theta_{s}C_{j}\right)+\nabla \cdot \left(\theta_{s}J_{j}\right)=R_{j},$$

where $R_{j}$ is the rate of change of dissolved ion j’s concentration per total volume due to all chemical reactions which produce/remove these ions. We assume that the chemical reactions which affect ion j are binding to and dissociation from the binding sites present on the network. These reactions are governed by the law of mass action, scaled appropriately with the heterogeneous volume in which the species are dissolved (see [17])

(56) $$R_{j}=R_{j}^{\mathrm{off}}-R_{j}^{\mathrm{on}}=k_{j}^{\mathrm{off}}B_{\mathrm{Mj}}\theta_{s}^{2}-k_{j}^{\mathrm{on}}m\theta_{s}C_{j},$$

where *m* is given in Equation (51). $k_{j}^{\mathrm{on}}$ and $k_{j}^{\mathrm{off}}$ are the binding and unbinding rate coefficients for ion j. Equation (55) together with Equation (5) gives (after some rearrangement) the transport equation

(57) $$\frac{\partial C_{j}}{\partial t}+\nabla \cdot \left(u_{s}C_{j}\right)=\frac{1}{\theta_{s}}\nabla \cdot \left(\theta_{s}D_{j}\left(\nabla C_{j}+z_{j}C_{j}\nabla \Psi \right)\right)-k_{j}^{\mathrm{on}}mC_{j}+k_{j}^{\mathrm{off}}B_{\mathrm{Mj}}\theta_{s}.$$

In a system with dissolved ions Na, Ca, and Cl (where Cl anions are assumed to be unable to bind/unbind with the network) this yields the equations

(58) $$\frac{\partial C_{\mathrm{Na}}}{\partial t}+\nabla \cdot \left(u_{s}C_{\mathrm{Na}}\right)=\frac{1}{\theta_{s}}\nabla \cdot \left(\theta_{s}D_{\mathrm{Na}}\left(\nabla C_{\mathrm{Na}}+C_{\mathrm{Na}}\nabla \Psi \right)\right)-k_{\mathrm{Na}}^{\mathrm{on}}mC_{\mathrm{Na}}+k_{\mathrm{Na}}^{\mathrm{off}}B_{\mathrm{MNa}}\theta_{s},$$

(59) $$\frac{\partial C_{\mathrm{Ca}}}{\partial t}+\nabla \cdot \left(u_{s}C_{\mathrm{Ca}}\right)=\frac{1}{\theta_{s}}\nabla \cdot \left(\theta_{s}D_{\mathrm{Ca}}\left(\nabla C_{\mathrm{Ca}}+2C_{\mathrm{Ca}}\nabla \Psi \right)\right)-k_{\mathrm{Ca}}^{\mathrm{on}}mC_{\mathrm{Ca}}+k_{\mathrm{Ca}}^{\mathrm{off}}B_{\mathrm{MCa}}\theta_{s},$$

(60) $$\frac{\partial C_{\mathrm{Cl}}}{\partial t}+\nabla \cdot \left(u_{s}C_{\mathrm{Cl}}\right)=\frac{1}{\theta_{s}}\nabla \cdot \left(\theta_{s}D_{\mathrm{Cl}}\left(\nabla C_{\mathrm{Cl}}-C_{\mathrm{Cl}}\nabla \Psi \right)\right).$$

The bound ionic species are assumed to advect with the network velocity and to not diffuse. The electrical forces acting on the charges in the network contribute to determining the network velocity $u_{n}$ as seen in Equation (33). Then, conservation of mass gives the following equations for the evolution of the bound ion concentrations

(61) $$\frac{\partial B_{\mathrm{MNa}}}{\partial t}+\nabla \cdot \left(u_{n}B_{\mathrm{MNa}}\right)=k_{\mathrm{Na}}^{\mathrm{on}}mC_{\mathrm{Na}}\theta_{s}-k_{\mathrm{Na}}^{\mathrm{off}}B_{\mathrm{MNa}}\theta_{s}^{2},$$

(62) $$\frac{\partial B_{\mathrm{MCa}}}{\partial t}+\nabla \cdot \left(u_{n}B_{\mathrm{MCa}}\right)=k_{\mathrm{Ca}}^{\mathrm{on}}mC_{\mathrm{Ca}}\theta_{s}-k_{\mathrm{Ca}}^{\mathrm{off}}B_{\mathrm{MCa}}\theta_{s}^{2}+2k_{\mathrm{Ca}}^{\mathrm{off}}B_{M_{2}\mathrm{Ca}}-\frac{1}{2}k_{\mathrm{Ca}}^{\mathrm{on}}mB_{\mathrm{MCa}},$$

(63) $$\frac{\partial B_{M_{2}\mathrm{Ca}}}{\partial t}+\nabla \cdot \left(u_{n}B_{M_{2}\mathrm{Ca}}\right)=-2k_{\mathrm{Ca}}^{\mathrm{off}}B_{M_{2}\mathrm{Ca}}+\frac{1}{2}k_{\mathrm{Ca}}^{\mathrm{on}}mB_{\mathrm{MCa}}.$$

Details regarding the reaction rate scalings are given in [17].

Electroneutrality ensures that the concentrations of charged species (measured in moles per *total volume*) sum to zero. This may be expressed as

(64) $$B_{\mathrm{MNa}}+2B_{\mathrm{MCa}}+2B_{M_{2}\mathrm{Ca}}-m^{\mathrm{tot}}\theta_{n}+\theta_{s}\left(C_{\mathrm{Na}}+2C_{\mathrm{Ca}}-C_{\mathrm{Cl}}\right)=0.$$

The electric potential gradient ∇Ψ does not have a constitutive equation, but may rather be viewed as a Lagrange multiplier whose role is to enforce the algebraic constraint Equation (64).

## 3. Solution Strategy

Equations (4)–(6), (49) and (50), and (58)–(64) represent the model equations we solve numerically. These equations form a coupled nonlinear system of PDEs (of mixed type) with algebraic and incompressibility constraints. All spatial derivative terms are discretized using finite-difference approximations. A fractional step scheme is used for time iteration from $t^{k}$ to $t^{k+1}$ as follows:

1. For given ion concentrations, electrical potential Ψ and volume fractions ($\theta_{s}$ and $\theta_{n}$) at time level $t^{k}$, compute all chemical forces appearing in (49) and (50). Solve the discretized version of (49) and (50), together with the incompressibility condition (6) to obtain the velocities for the network and solvent $u_{n}$ and $u_{s}$ at $t^{k}$.
2. Extrapolate $u_{n}$ and $u_{s}$ to the half time step $t^{k+1/2}$ from their values at $t^{k-1}$ and $t^{k}$. Based on the extrapolated velocities, solve the discretized version of (4) to get the updated values for $\theta_{n}$ and $\theta_{s}$ at $t^{k+1}$. Similarly, update all ion concentrations to account for their advective transport using a discretized version of (58)–(63).
3. Update the concentrations of bound ions at $t^{k+1}$ to account for all reaction terms in discretized versions of (61)–(63).
4. Update the concentrations of dissolved ions and the electric potential Ψ at $t^{k+1}$ to account for the diffusive transport, electromigration, and reaction in discretized versions of (58)–(60) and (64).

A MAC-type staggered computational grid is used for the spatial discretization, where scalars are located at the grid centers and vectors are located at the centers of grid edges. In step 1, all spatial derivatives in the equations are approximated using second-order, centered difference formulas. The discrete equations form a large, sparse nonsymmetric linear system of saddle point type. The system is solved by a GMRES solver, which is preconditioned by a multigrid scheme [24]. In step 2, the advective terms are discretized using the second-order, unsplit Godunov Scheme, as described in [25]. In step 3, all reaction terms are discretized implicitly. The resulting 3×3 linear system for each grid cell is solved directly. Finally, in step 4, the sparse linear system from the discretization is solved by a multigrid-preconditioned GMRES solver. Details about the algorithm can be found in [20]. In our simulations of gel swelling, the network volume fraction is zero in parts of the domain. To avoid a degenerate network Equation (49), numerical regularization as proposed in [26] is used for the solution of fluid velocities in step 1.

## 4. Results

### 4.1. Problem Setup

The values of model constants are list in Table 1.

In the table, $\nu_{n}$ is computed based on the monomer molarity $m^{\mathrm{tot}}=0.1$ M. The drag coefficient is set to $\xi /\nu =\frac{\eta_{s}}{L_{0}^{2}}$ [29], where $L_{0}=40$ nm is the characteristic pore size of a mucin gel [30]. We choose the values of the interaction energy so that the Flory–Huggins parameter χ(α) is an increasing linear function of the cross-link fraction α. Based on this choice, a fully cross-linked network with α=1 has χ=0, so short-range interactions make no contribution to swelling. For a network without any cross-links and α=0, χ=−18 and the interaction energy promotes rapid swelling. We choose $\epsilon_{\mathrm{pp}}-\epsilon_{\mathrm{uu}}=0$ and $\epsilon_{\mathrm{xx}}-\epsilon_{\mathrm{uu}}=-0.5$ so that $\epsilon_{\mathrm{xx}}$ is only slightly lower than $\epsilon_{\mathrm{pp}}$ and $\epsilon_{\mathrm{uu}}$. A more negative value of $\epsilon_{\mathrm{xx}}-\epsilon_{\mathrm{uu}}$ tends to drive deswelling of the gel, which is not the focus of this study. Our simulation setup is intended to mimic the swelling of a blob of highly cross-linked gel immersed in a fluid solvent of known dissolved ion composition.

**Figure 1:**
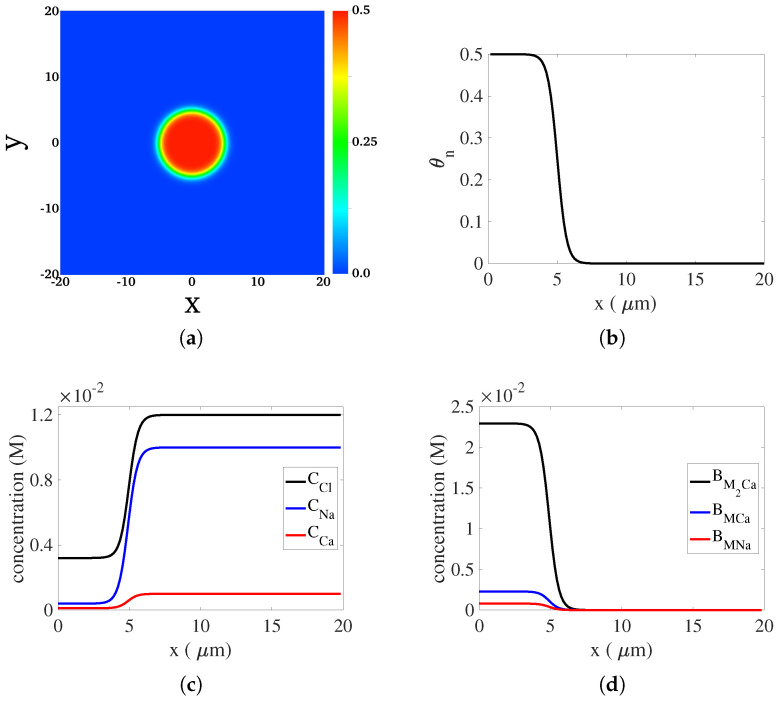
The two-dimensional computational domain and the initial distributions of model variables. (**a**) Initial distribution of $\theta_{n}$. (**b**) Profile of $\theta_{n}$ at t=0 along the positive x-axis. (**c**) Profiles of the concentrations for dissolved ions at t=0 along the positive x-axis. (**d**) Profiles of the concentrations of bound ions at t=0 along the positive x-axis.

As shown in Figure 1a, the computational domain is the two-dimensional region (x,y)∈[−20μm,20μm]×[−20μm,20μm]. At t=0, there is a circular region at the center of the domain with an appreciable amount of network. We refer to this region as the “gel”. We refer to the rest of the domain as the “bath”. Typical initial profiles of $\theta_{n}$, and the concentrations of dissolved and bound ions along the positive x-axis are shown in Figure 1b–d. For a specific model variable, its initial value inside the gel is set to a constant in the bulk of the gel. This uniform profile of the variable in the gel transitions smoothly but sharply to a different uniform profile in the bath.

To construct the initial conditions for the ion concentrations in the gel region, we prescribe uniform values for $\theta_{n}$, $m^{\mathrm{tot}}$ and the total concentrations (bound plus dissolved) of sodium, calcium, and chloride ions in that region. These concentrations are chosen so that net charge at each point is zero. We then determine the bound and dissolved ion concentrations by computing the steady-state solution of the ODEs that result from Equations (58)–(63) when the spatial profiles are assumed to be uniform so that all of the transport terms vanish. These solutions are the initial concentrations we use within the gel. For most of the bath region, $\theta_{n}=0$; there is a sharp but smooth connection at the edge of the gel between the value of $\theta_{n}$ in the bulk of the gel and the zero values in most of the bath. We specify the concentrations of dissolved ions in the bath and set the concentration of bound ions there to zero. By the way that we select the initial concentrations, there is a separate chemical equilibrium in the bulk of the gel and in the bulk of the bath. In this paper, we set $k_{\mathrm{Na}}^{\mathrm{on}}$ and $10^{6}$ M${}^{-1}\;s^{-1}$, $k_{\mathrm{Na}}^{\mathrm{off}}=10^{3}$ $s^{-1}$, $k_{\mathrm{Ca}}^{\mathrm{on}}=5\times 10^{6}$ M${}^{-1}\;s^{-1}$, and $k_{\mathrm{Ca}}^{\mathrm{off}}=5\times 10^{2}\;s^{-1}$. Defining the dissociation constant of ion j as $K_{j}^{D}=k_{j}^{\mathrm{off}}/k_{j}^{\mathrm{on}}$, we have $K_{\mathrm{Ca}}^{D}<K_{\mathrm{Na}}^{D}$ and thus calcium binding to the network is chemically preferred.

### 4.2. Observed Swelling Dynamics

**Figure 2:**
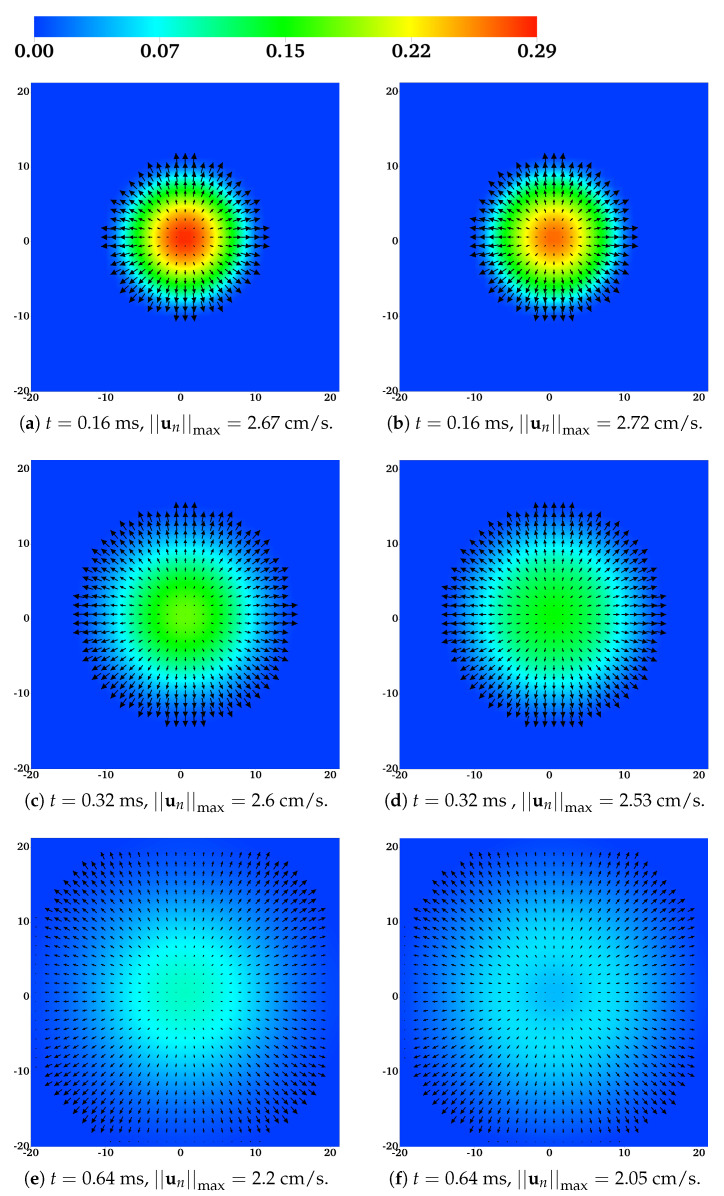
The distribution of the network volume fraction $\theta_{n}$ and the network velocity field $u_{n}$ at different times for (**a**,**c**,**e**) $C_{\mathrm{Na}}^{*}=$ 0.001 M and (**b**,**d**,**f**) $C_{\mathrm{Na}}^{*}=$ 0.05 M. All vectors have the same scale.

The initial conditions are given in Table 2. We choose total ion concentrations so that chemical equilibrium within the gel gives the cross-link fraction α≈0.92. We vary the value of $C_{\mathrm{Na}}$ in the bath to see the effects on swelling dynamics. In Figure 2a,c,e and Figure 2b,d,f, we plot the distributions at times t= 0.16, 0.32, and 0.64 ms of $\theta_{n}$ and $u_{n}$ for the sodium bath concentrations, $C_{\mathrm{Na}}^{*}=0.001$ M and $C_{\mathrm{Na}}^{*}=0.05$ M, respectively. The velocity field is roughly radially symmetric and the largest magnitude network velocities occur along the edge of the gel and decrease monotonically towards the gel center. The maximum velocity decreases with time for both sodium bath concentrations, and it does so more rapidly in the high-sodium case. Relative to the initial profile shown in Figure 1a, $\theta_{n}$ in Figure 2 becomes progressively more homogeneous in space as time advances and the network moves out from the center of the domain. The simulation with the higher value of $C_{\mathrm{Na}}^{*}$ has a more uniform network distribution at the final time pictured.

**Figure 3:**
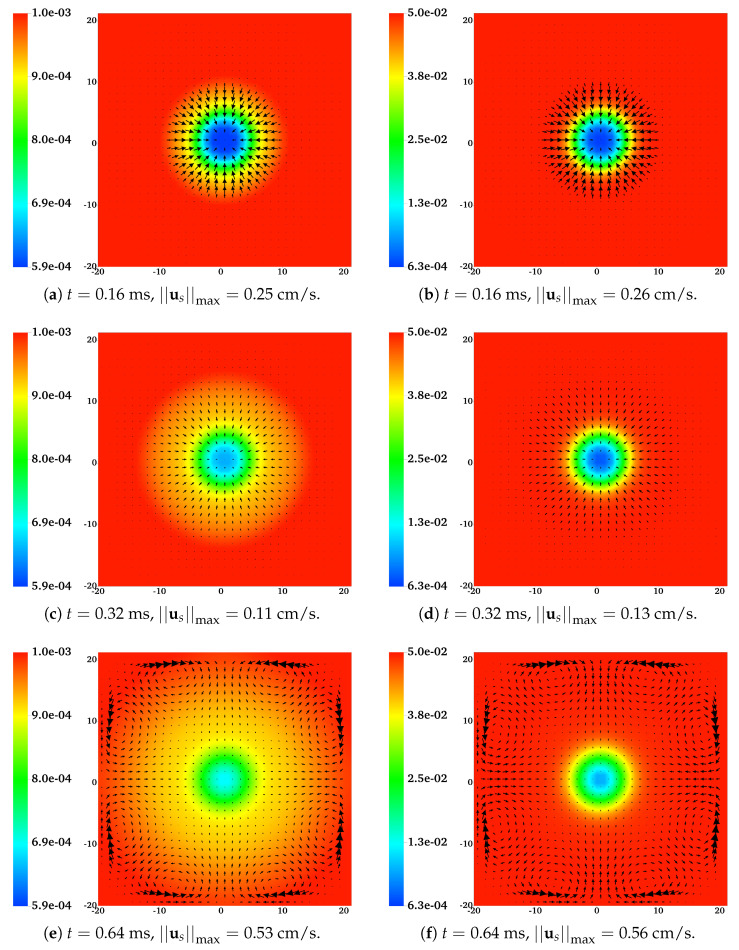
The concentration of dissolved sodium ions $C_{\mathrm{Na}}$ and the solvent velocity field $u_{s}$ at different times for (**a**,**c**,**e**) $C_{\mathrm{Na}}^{*}=$ 0.001 M and (**b**,**d**,**f**) $C_{\mathrm{Na}}^{*}=$ 0.05 M. All vectors have the same scale.

The distributions of $C_{\mathrm{Na}}$ and $u_{s}$ at t= 0.16, 0.32, 0.64 ms for the two simulations are shown in Figure 3. For both the low-sodium case (Figure 3a,c,e) and the high-sodium one (Figure 3b,d,f), we can see significant inward movement of the solvent. The magnitude of the solvent velocity varies non-monotonically with distance from the gel center at x=0. At each time, it is highest at a distance roughly midway between the center and edge of the gel. At the edge of the gel, the magnitude of $u_{s}$ is much smaller than that for $u_{n}$ because $\theta_{n}\ll \theta_{s}$ at that location. At the final time shown, the solvent velocity is also not radially symmetric because of the no-slip boundary conditions that hold on the domain boundary. The inward movement of solvent carries dissolved sodium ions from the bath into the gel and contributes to an increase of $C_{\mathrm{Na}}$ there.

We seek a concise measure of swelling extent, and apply it to simulations done with three different values of $C_{\mathrm{Na}}^{*}$, namely, $C_{\mathrm{Na}}^{*}=0.001$ M, $C_{\mathrm{Na}}^{*}=0.01$ M, and $C_{\mathrm{Na}}^{*}=0.05$ M, which for brevity we refer to as the ‘low-sodium’, ‘medium-sodium’, and ‘high-sodium’ cases, respectively. Because of the approximate radial geometry of the gel, its swelling is not adequately conveyed by plots of the distribution of $\theta_{n}$ along a line. To better quantify the extent of gel swelling, we define the *cumulative network volume fraction function*.

(65) $$\beta \left(r\right)=\frac{\int_{\Omega_{1}\left(r\right)}\theta_{n}dS}{\int_{\Omega }\theta_{n}dS}.$$

**Figure 4:**
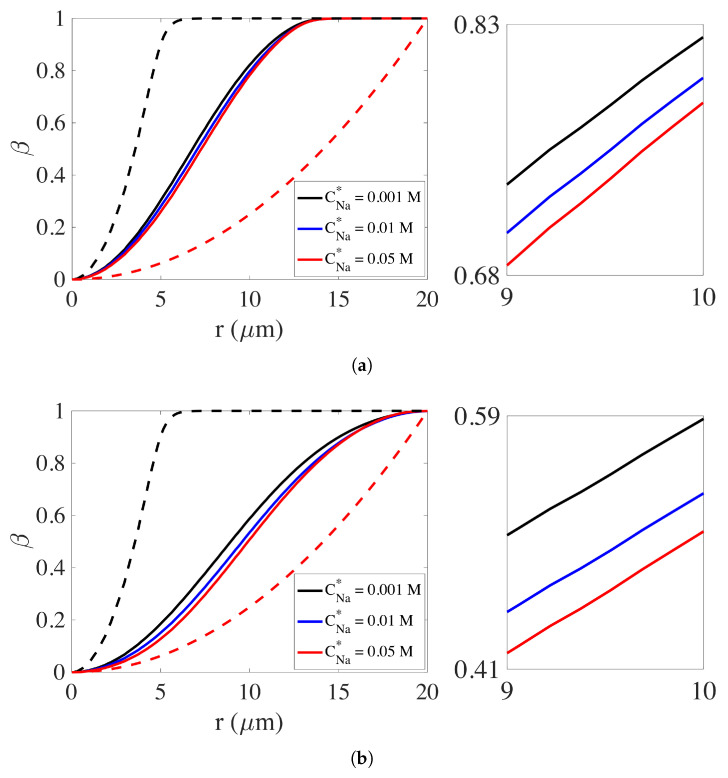
The cumulative network volume fraction function β(r), defined in Equation (65), for simulations with $C_{\mathrm{Na}}^{*}=$ 0.001 M, 0.01 M, and 0.05 M. The dashed black curve is for the initial profile. The dashed red curve is for a uniform distribution of network in a disk of radius 20 μm. (**a**) t=0.32 ms. (**b**) t=0.56 ms.

Here r is the radial distance from the origin, $\Omega_{1}\left(r\right)$ is the disk of radius r centered at the origin, and Ω is the whole computational domain. The value of β(r) represents the fraction of the total network that is inside the disk of radius r. In Figure 4, we show plots of β(r) at t=0.32 ms and t=0.56 ms for simulations with different $C_{\mathrm{Na}}^{*}$. The latter time is chosen as the moment when the edge of the gel approximately reaches the domain boundary for all three simulations. In the plot, the dashed black curve is the profile of β(r) at t=0. The dashed red curve shows β(r) if the network were uniformly distributed over the disk with radius 20μm. From the plots at t=0.32 ms, we see that with the increase in the sodium bath concentration, the curves of β(r) become closer to that for the uniform distribution. In other words, the gel has expanded more and become more homogeneous in space the larger the value of $C_{\mathrm{Na}}^{*}$. Comparison of the plots in Figure 4b for t=0.56 ms with those at t=0.32 ms in Figure 4a shows the progression of swelling. At t=0.32 ms, β(r) reaches very close to 1 by r≈15μm which indicates that essentially all of the network is contained inside a disk of radius 15 μm, while for the later time it is not until *r* is close to 20 μm that β(r) approaches 1. At the later time, the differences in the extent of swelling for the different values of $C_{\mathrm{Na}}^{*}$ becomes clearer. The right parts of Figure 4a,b show blowups of portions of the β(r) curves, and from them we can see clearly the monotonic increase in overall swelling speed as $C_{\mathrm{Na}}^{*}$ is increased.

### 4.3. Analysis of the Swelling Behaviors

The different swelling behaviors, that is, the different network and solvent relative motions, and how they are influenced by bath concentrations, seen in the experiments are a result of changes in the relative magnitudes and directions of forces acting on the two materials. Six types of forces act on each of the network and solvent; these include the three chemical forces arising respectively from short-range, entropic, and electrical interactions and the three mechanical forces due to viscosity within each material, drag between the network and solvent, and pressure. The viscous and drag forces arise in response to relative motions and act to dampen them. They can transfer momentum but do not otherwise initiate movement. The pressure and electrical forces enforce, respectively, the constraints that $\theta_{n}u_{n}+\theta_{s}u_{s}$ must be incompressible and that electroneutrality must always be maintained. They arise when motions due to other forces would otherwise cause these constraints to be violated. The main driving forces for the motions are the short-range interaction and entropic forces, the other forces modulate the response to these forces and thereby help shape the overall motions. In this section, we examine these forces, how they influence and are influenced by the distribution of particles and charges, and how they are affected by the chemistry of ion binding/unbinding with the network.

**Figure 5:**
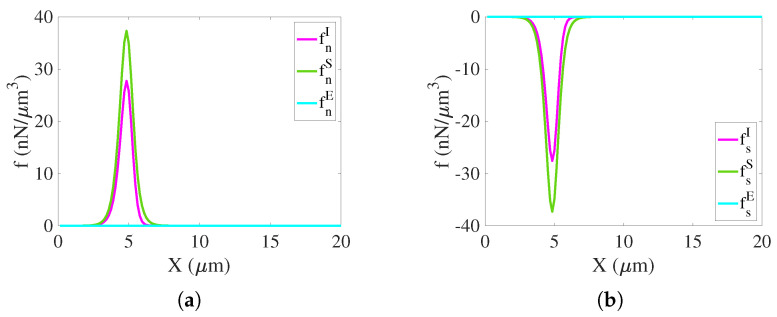
Chemical force densities on the network and solvent at t=0. (**a**) chemical force on network. (**b**) chemical force on solvent.

**Initial chemical force densities**. In Figure 5 we plot the initial chemical force density components $f_{n}^{I}$, $f_{n}^{S}$, and so on, which act on the network and solvent. At this time, the entropic and short-range interaction force densities, which are nonzero near the initial edge of the gel, contribute to initiate swelling. The electrical force densities $f_{n}^{E}$ and $f_{s}^{E}$ are zero at t=0.

**Figure 6:**
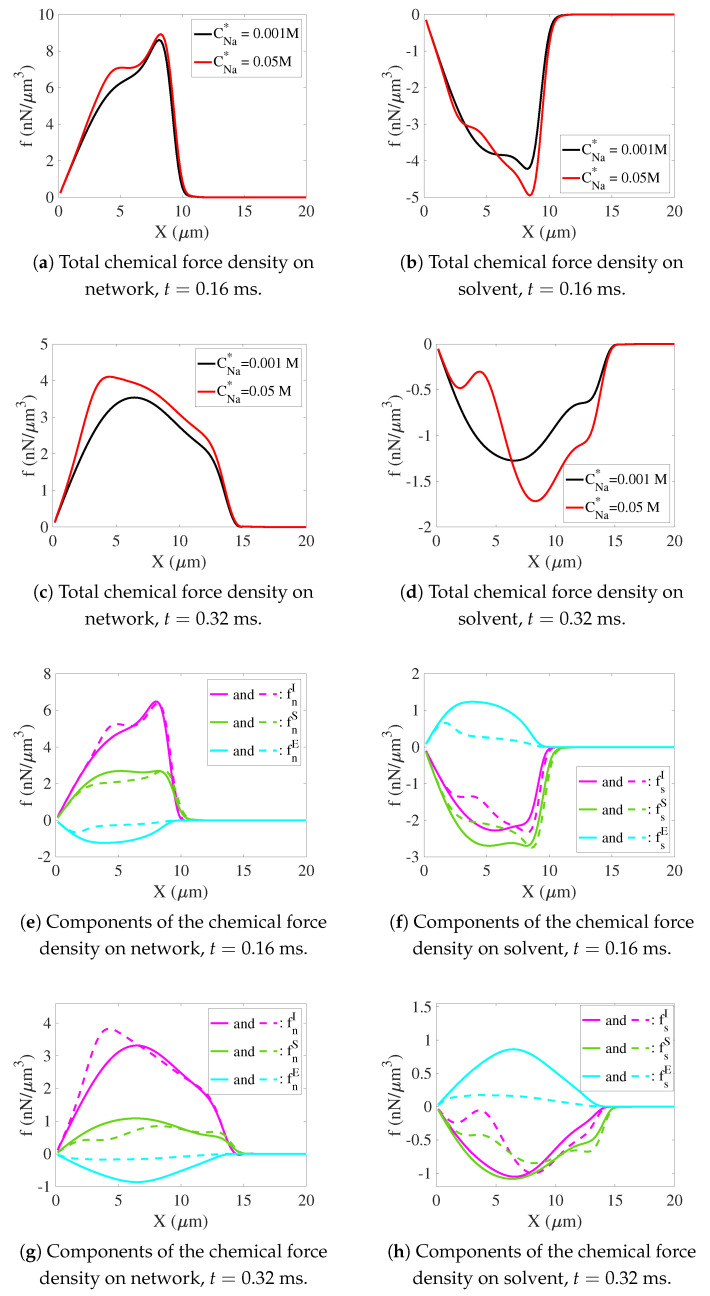
Network and solvent chemical force densities along the positive x-axis. Total chemical force densities on network and solvent at (**a**,**b**) t=0.16 ms and (**c**,**d**) t=0.32 ms. Components of the network and solvent chemical force densities for $C_{\mathrm{Na}}^{*}=0.001$ M (solid) and $C_{\mathrm{Na}}^{*}=0.05$ M (dashed) at (**e**,**f**) t=0.16 ms and (**g**,**h**) t=0.32 ms.

**Total chemical and mechanical force densities**. In Figure 6, we plot the variation of the chemical force densities along the positive x-axis for the low- and high-sodium cases. By symmetry, all of these force densities are in the x-direction. From Figure 6a,c, we see that there is a large positive chemical force density on the network which is the driving force for the network’s swelling. This force density is larger for the high-sodium case and becomes progressively more so as time advances. The differences are largest close to the center of the gel (3<x<6). In Figure 6b,d, we see that the total chemical force density on the solvent acts in the negative x-direction, and that, at t=0.32 ms, the changes in this total force density when the sodium bath concentration is increased, are more complicated than is the change in the total network force density. With the increase in $C_{\mathrm{Na}}^{*}$, the chemical force density on the solvent pushing it towards the center of the gel decreases in strength for 0<x<6 and increases in strength in the remainder of the gel, 6<x<15. Since there is a local force balance in both the network and the solvent at every point, the total mechanical force density on each of the network and solvent must balance the total chemical force densities just described.

**Chemical force density components**. The components of the chemical force densities on the network and solvent at t= 0.16 ms and 0.32 ms are shown, respectively, in Figure 6e,g and Figure 6f,h. By examining these force density components and what other changes between the low- and high-sodium cases affected them, we seek to explain the greater swelling tendency of the high-sodium case.

**Overview of chemical force components**. For both sodium bath concentrations, the short-range and entropic force densities on the network act in the positive x-direction, consistent with the direction of gel swelling, and the electrical force density on the network acts in the negative x-direction, thus opposing its outward motion. For the solvent, the short-range and entropic force densities act in the negative x-direction and thus contribute to swelling by allowing solvent to displace network within the gel. The electrical density force acts in the positive x-direction and thus opposes swelling. For x<6, the larger chemical force density on the network for the high-sodium case is due to a combination of a larger positive short-range interaction force density and a smaller magnitude negative electrical force density, as well as to a somewhat less positive entropic force density for x<8. For 6<x, the larger chemical force density on the network is due primarily to a less negative electrical force density. For the solvent, the higher sodium bath concentration results in the short-range interaction force density and the entropic force density on the solvent becoming less negative for x<7 while they are largely unchanged for larger x. The positive electrical force density on the solvent decreases throughout the gel (0<x<15). The net effect is a less negative total chemical force density on the solvent for small x and the more negative one for large x shown in Figure 6d.

**Figure A1:**
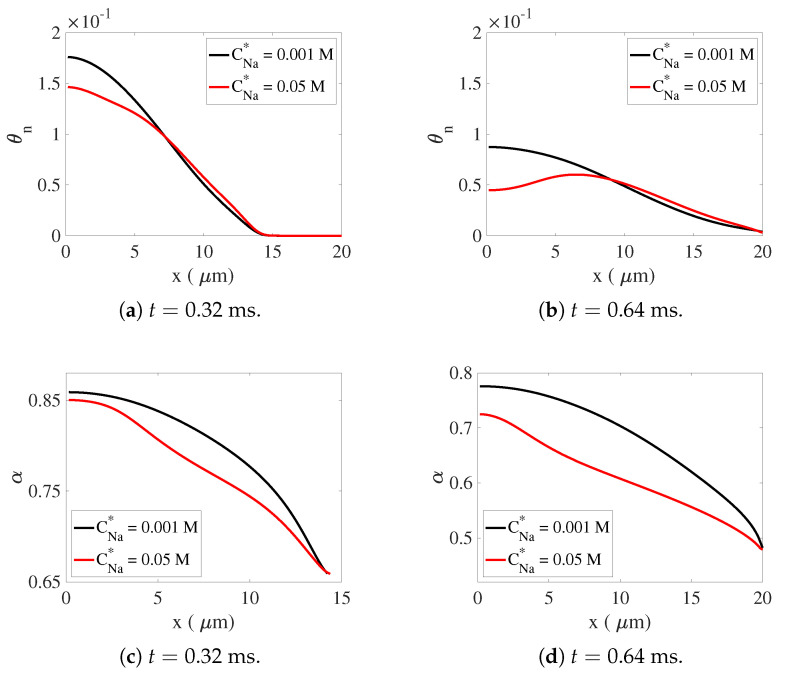
Simulations with sodium bath concentrations $C_{\mathrm{Na}}^{*}=0.001$ M and 0.05 M. Distributions of the network volume fraction $\theta_{n}$ along the positive x-axis at (**a**) t=0.32 ms and (**b**) t=0.64 ms. Distributions of the network cross-link fraction α at (**c**) t=0.32 ms and (**d**) t=0.64 ms.

**Entropic force density**. The entropic forces are the easiest to understand. The entropic force density on the network given in Equation (35) always pushes the network down the gradient of the network volume fraction and has magnitude that increases with the magnitude of $\nabla \theta_{n}$ and with the value of $\theta_{n}$ itself. Hence, the lower value of $\theta_{n}$ for x<7 and the less steep gradient in $\theta_{n}$ for x<8, shown in Figure A1a for the high-sodium case explains the smaller entropic force on the network in that case. For x>8, the profiles of $\theta_{n}$ are very similar for the two simulations and the corresponding entropic force densities on the network are almost the same. The entropic force density on the solvent is equal and opposite to that for the network and therefore changes in it are also always equal and opposite to those on the network.

**Network short-range force densities**. As seen in Figure 6e,g, the short-range interaction force densities on the network are large and positive for both sodium bath concentrations, and significantly larger near the gel’s center for the high-sodium case at t=0.32 ms. From Equation (47), we calculate the short-range interaction force density acting on the network to be

(66) $$f_{n}^{I}=-\frac{k_{B}T}{\nu_{n}}\left\{\chi \left(\alpha \right)\theta_{n}\theta_{s}\nabla \theta_{s}+\theta_{n}\left(\frac{1}{2}\theta_{s}^{2}\chi^{\prime }\left(\alpha \right)+{\left(\mu_{n}^{0}\left(\alpha \right)\right)}^{\prime }\right)\nabla \alpha \right\},$$

where ${}^{\prime }$ indicates differentiation with respect to α. From this formula, we see that the short-range interaction forces depend on the magnitudes of the network volume fraction $\theta_{n}$ and the cross-link fraction α as well as their respective spatial gradients. Using the parameter values from Table 1, we find that $\chi^{\prime }\left(\alpha \right)=18$ and ${\left(\mu_{n}^{0}\right)}^{\prime }\left(\alpha \right)=-1/4$, and so we can write

(67) $$f_{n}^{I}=\frac{k_{B}T}{\nu_{n}}\left\{\chi \left(\alpha \right)\theta_{n}\left(1-\theta_{n}\right)\nabla \theta_{n}-\theta_{n}\left(9\theta_{s}^{2}-1/4\right)\nabla \alpha \right\}$$

where χ(α) ranges from 0 for α=1 to −18 for α=0. There is a contribution in the direction opposite that of $\nabla \theta_{n}$ and another contribution in the direction of lower values of α (unless $\theta_{s}^{2}<1/36$). The first of these contributions has magnitude which grows rapidly with the decreases of α that occur when cross-links break, and progressively decreases as $\theta_{n}$ moves from 1/2 toward 0, that is, as swelling proceeds. The second contribution has magnitude proportional to the local network volume fraction and to the magnitude of the gradient of α. In the setting of our experiments, both terms contribute to push the network toward larger values of x, that is, to swell. In Figure A1a we see that both $\theta_{n}$ and the size of its gradient are smaller for the high-sodium case for x<7, and from Figure A1b, we see that α is lower and decreases more rapidly with x for high sodium. The differences in the behavior of the cross-link fraction α, including a significantly more negative value of χ(α) in the first term in the expression for $f_{n}^{I}$ in Equation (67) and the larger gradient in the second term, more than compensate for the changes in $\theta_{n}$ to produce a greater short-range force for the high-sodium case.

**Figure A2:**
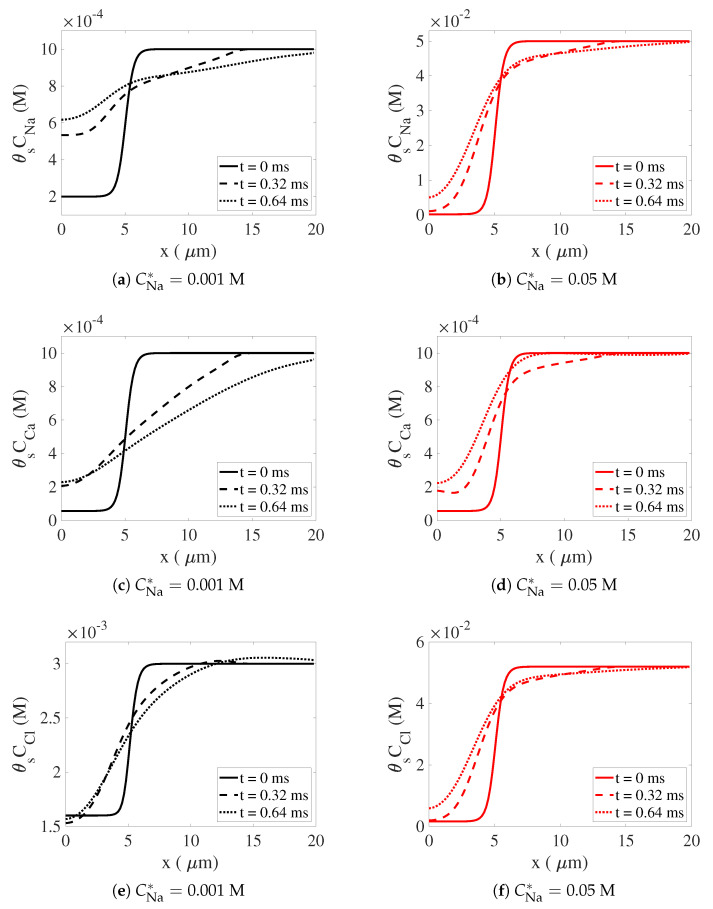
Simulations with sodium bath concentrations (**a**,**c**,**e**) $C_{\mathrm{Na}}^{*}=0.001$ M and (**b**,**d**,**f**) $C_{\mathrm{Na}}^{*}=0.05$ M. Distribution of dissolved ions along the positive x-axis (concentrations per total volume) at t=0, 0.32, and 0.64 ms for (**a**,**b**) sodium, (**c**,**d**) calcium, and (**e**,**f**) chloride.

**Figure A3:**
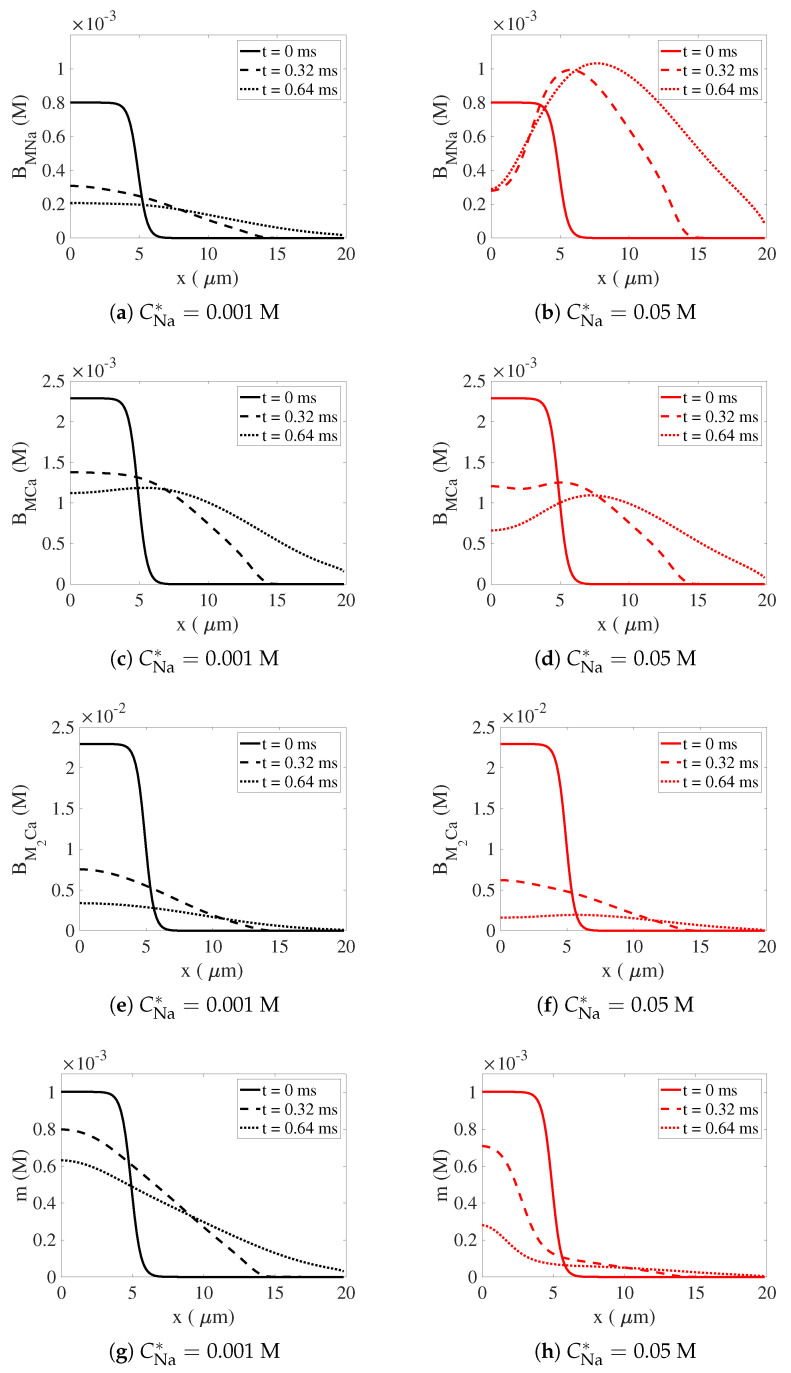
Simulations with sodium bath concentrations (**a**,**c**,**e**) $C_{\mathrm{Na}}^{*}=0.001$ M and (**b**,**d**,**f**) $C_{\mathrm{Na}}^{*}=0.05$ M. Distribution of bound ions along the positive x-axis (concentrations per total volume) at t=0, 0.32, and 0.64 ms for (**a**,**b**) sodium, (**c**,**d**) calcium bound to one network monomer, and (**e**,**f**) calcium cross-linking two network monomers, and (**g**,**h**) unoccupied network binding sites. Note that only calcium bound to one network monomer and unoccupied network binding sites carry charge, a single positive or negative charge, respectively.

**Dynamics of α and the bound ions**. The reasons for the different dynamics of the cross-link fraction α for the different sodium bath concentrations can be traced to differences in the dissolved and bound ion distributions shown in Figure A2 and Figure A3, respectively. With the higher sodium bath concentration, there is a much larger flux of sodium ions from the bath into the gel. This greatly increases $C_{\mathrm{Na}}$ within the gel, and also leads to a large increase in the concentration of network-bound sodium ions $B_{\mathrm{MNa}}$. The relative size of the dissolved sodium ion and dissolved calcium ion concentrations in the high-sodium case allows for greater binding of sodium to the network even though we have assumed that calcium binds more tightly to the network than does sodium, i.e., $K_{\mathrm{Ca}}^{D}<K_{\mathrm{Na}}^{D}$. As a consequence of the greater sodium binding, the concentration of unoccupied binding sites on the network *m* (Figure A3g,h) is greatly reduced and this reduces the opportunities for dissolved calcium and singly-bound calcium ions to bind to the network. Because of this, the density of doubly-bound calcium ions is lower for the high-sodium case (see Figure A3e,f), which results in the lower network cross-link fraction that we discussed above.

**Solvent short range force**. The short-range interaction force densities on the solvent are shown in Figure 6f,h. Those in the high-sodium case are smaller in magnitude for 1<x<8 and elsewhere approximately the same compared to the force densities for the low-sodium case. From Equation (48), and using the parameter values from Table 1, we find that

(68) $$f_{s}^{I}=\frac{k_{B}T}{\nu_{n}}\left\{-\chi \left(\alpha \right)\theta_{n}\left(1-\theta_{n}\right)\nabla \theta_{n}-9\theta_{n}^{2}\left(1-\theta_{n}\right)\nabla \alpha \right\}.$$

In the first term, χ(α)<0 and $\nabla \theta_{n}<0$, so this term contributes a force on the solvent towards the center of the gel, while the second term contributes a force in the direction of lower α, that is away from the gel’s center. As a result of the opposing effects of these two terms of $f_{s}^{I}$, the effects of the short-range interaction forces on the solvent’s motion are complex.

**Figure 7:**
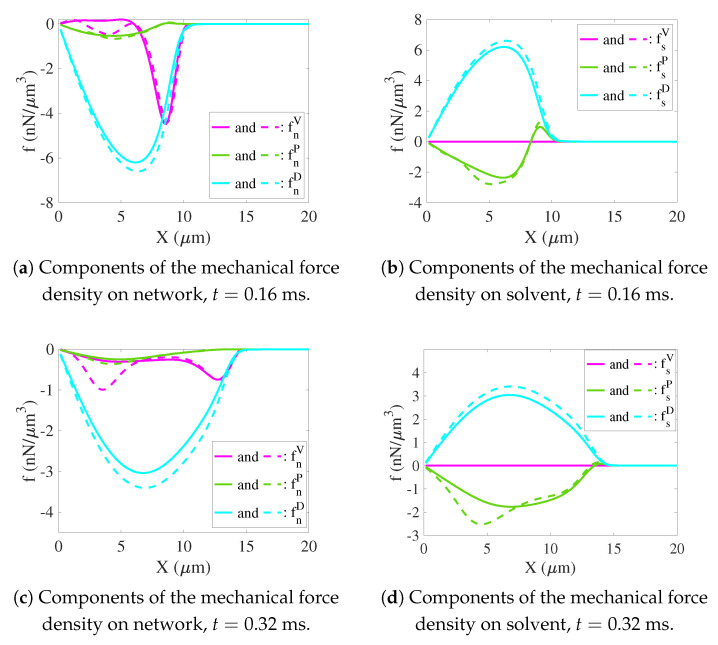
Components of the network and solvent mechanical force densities along the positive x-axis for $C_{\mathrm{Na}}^{*}=0.001$ M (solid) and $C_{\mathrm{Na}}^{*}=0.05$ M (dashed) at (**a**,**b**) t=0.16 ms and (**c**,**d**) t=0.32 ms. Here $f_{i}^{V}$, $f_{i}^{P}$, and $f_{i}^{D}$ represent the densities of viscous force, pressure force, and drag force on phase i (i=s,n), respectively.

**Balance between chemical and mechanical forces**. Since there is a local force balance in both the network and the solvent individually at every point, the total mechanical force density must balance the sum of the chemical force densities just described. From Figure 7a,c, we see that all of the components of the mechanical force on the network are negative. These forces are dominated by the drag forces which change little between the simulations with low and high sodium. The pressure forces on the network are very small and also differ little for the two sodium bath concentrations. The viscous forces are also very small except for the interval 6<x<10 for both sodium bath concentrations at t=0.16 ms and for the interval 8<x in the low sodium case, and in this interval as well as and the interval 2<x<6 for the high-sodium case at t=0.32 ms. In Figure 7b,d, we see that, at both t=0.16 ms and t=0.32 ms, there is a large positive drag force on the solvent which changes little with the sodium bath concentration, there is an extremely small viscous force, and there is a moderately large and negative pressure force which becomes significantly more negative for x<7 as the sodium bath concentration is increased.

**Electrical force densities and electromigration**. As discussed above, in the case of the higher sodium bath concentration, the larger influx of sodium into the gel leads to a faster decrease in the cross-link fraction α and thereby to larger short-range interaction forces that promote faster swelling. The larger sodium influx also leads to substantially different distributions of ions within the solvent and different net charges on each of the network and solvent. These affect the magnitude of the electrical force densities which act on the network and solvent and also influence the magnitude, distribution, and nature of the electromigration fluxes of dissolved ions needed to maintain electroneutrality.

The difference in ion distributions can be seen in the plots of dissolved ion concentrations in Figure A2 and bound ion concentrations in Figure A3. The left and right columns of Figure A2 show the variations along the positive x-axis of the total concentrations $\theta_{s}C_{j}$ (amount per total volume) of sodium, calcium, and chloride ions initially and at times t=0.32 ms and t=0.64 ms for the low- and high-sodium cases, respectively. Comparing the concentrations in the high-sodium case with the corresponding ones in the low-sodium case, we see that (i) all of the dissolved ion concentrations are higher, with those of sodium and chloride being much higher, and (ii) the gradients of the concentrations are much larger, in particular in the center of the gel (0<x<6). In Figure A3, we plot the concentrations of ions bound to the network and the concentration *m* of unoccupied negatively charged sites on the network. In Figure A3e,f we see that $B_{M_{2}\mathrm{Ca}}$ is lower for the high-sodium case than for the low one, consistent with the greater decrease in the cross-link fraction α, and the larger short-range interaction force in the high sodium case. We also see that $B_{\mathrm{MCa}}$ is somewhat lower in the high-sodium case, which lowers the positive charge carried by the network. Most striking, however, is the much larger bound sodium concentration $B_{\mathrm{MNa}}$ and the correspondingly much lower unoccupied site concentration *m*, especially for 3<x, as a result of dissolved sodium binding to the network. The occupation of sites that would otherwise contribute a negative charge to the network pushes the net charge on the network to be much more positive in the high-sodium case and, simultaneously, makes the net charge on the solvent much more negative. These changes in the charge distribution between network and solvent have a strong effect on the electrical force densities $f_{n}^{E}$ and $f_{s}^{E}$. When combined with the relative motion of the network and solvent, the change in charge distribution also has major implications for the need for electromigration and the way that those needs are met.

Expressions for the electrical force density on the network, and the equal and opposite electrical force density on the solvent, are given in Equations (43) and (44). The electrical force densities along the positive x-axis on the solvent and network at t=0.16 ms and t=0.32 ms are shown in Figure 6e–h, respectively, and they both oppose swelling for both sodium bath concentrations. As is evident from that figure, these force densities are much larger for the low-sodium case and thus are a greater hindrance to swelling in that case. The electrical force densities are proportional to the net amounts of charge carried by the network and the solvent, as well as to the induced electric field −∇Ψ. As we have just discussed, the net negative charge on the solvent is greater in the high-sodium case, but, perhaps paradoxically, the electrical force density is much smaller in magnitude in that case, and, consequently, the electrical forces oppose swelling much less in the high-sodium case (compare Figure 6e,g with Figure 6f,h). While both a higher short-range force density and a lower magnitude electrical force density contribute to the larger total chemical force on the network for x<6, it is predominantly the lower-magnitude electrical force density that is responsible for the larger total positive chemical force on the network for 6<x<15.

To understand why the electrical forces oppose swelling more strongly in the low-sodium case, we examine the distribution of charges on the network and in the solvent as well as the factors contributing to alter and maintain the charge balance at each location. To that end, we define the net charge density at any location as

(69) $$P=\left(B_{\mathrm{MNa}}+2B_{\mathrm{MCa}}+2B_{M_{2}\mathrm{Ca}}-m^{\mathrm{tot}}\theta_{n}\right)+\theta_{s}\left(C_{\mathrm{Na}}+2C_{\mathrm{Ca}}-C_{\mathrm{Cl}}\right),$$

in which the charges on the network and the solvent appear in the first and second parenthetical terms on the right hand side, respectively. The value of *P* is affected by the spatial transport of all charged species by advection, diffusion, and electromigration, but is not directly affected by the reactions in which ions bind to or unbind from the network. These reactions shift charge between the phases, but not from one spatial location to another. The value of *P* must always be zero and so the contributions to its rate of change must sum to zero. To examine these contributions, we define the advection, diffusion, and electromigration *charge flux* vectors as

(70) $$J^{A}=u_{n}\left(B_{\mathrm{MNa}}+2\left(B_{\mathrm{MCa}}+B_{M_{2}\mathrm{Ca}}\right)-m^{\mathrm{tot}}\theta_{n}\right)+u_{s}\theta_{s}\left(C_{\mathrm{Na}}+2C_{\mathrm{Ca}}-C_{\mathrm{Cl}}\right),$$

(71) $$J^{D}=-\theta_{s}\left(D_{\mathrm{Na}}\nabla C_{\mathrm{Na}}+2D_{\mathrm{Ca}}\nabla C_{\mathrm{Ca}}-D_{\mathrm{Cl}}\nabla C_{\mathrm{Cl}}\right),$$

and

(72) $$J^{\mathrm{EM}}=-\theta_{s}\left(D_{\mathrm{Na}}C_{\mathrm{Na}}+4D_{\mathrm{Ca}}C_{\mathrm{Ca}}+D_{\mathrm{Cl}}C_{\mathrm{Cl}}\right)\nabla \Psi .$$

$J^{A}$ describes the net flux of charge due to the movement of the network and the solvent, and $J^{D}$ and $J^{\mathrm{EM}}$ describe analogous charge fluxes from diffusion and electromigration of dissolved ions, respectively. Each of these fluxes is calculated by multiplying the corresponding particle flux by that particle’s valence.

Each of these fluxes J contributes the quantity −∇·J to the rate of change of *P*. The contribution from advection is

(73) $$R^{A}=-\nabla \cdot J^{A}=-\nabla \cdot \left(u_{n}\left(B_{\mathrm{MNa}}+2B_{\mathrm{MCa}}+2B_{M_{2}\mathrm{Ca}})-m^{\mathrm{tot}}\theta_{n}\right)+u_{s}\theta_{s}\left(C_{\mathrm{Na}}+2C_{\mathrm{Ca}}-C_{\mathrm{Cl}}\right)\right),$$

and the contributions made by diffusion and electromigration of ions in the solvent are

(74) $$R^{D}=-\nabla \cdot J^{D}=\nabla \cdot \left(\theta_{s}\left(D_{\mathrm{Na}}\nabla C_{\mathrm{Na}}+2D_{\mathrm{Ca}}\nabla C_{\mathrm{Ca}}-D_{\mathrm{Cl}}\nabla C_{\mathrm{Cl}}\right),\right),$$

and

(75) $$R^{\mathrm{EM}}=-\nabla \cdot J^{\mathrm{EM}}=\nabla \cdot \left(\theta_{s}\left(D_{\mathrm{Na}}C_{\mathrm{Na}}+4D_{\mathrm{Ca}}C_{\mathrm{Ca}}+D_{\mathrm{Cl}}C_{\mathrm{Cl}}\right)\nabla \Psi \right),$$

respectively. We further decompose $J^{\mathrm{EM}}$ and $R^{\mathrm{EM}}$ into portions connected with the dissolved sodium, calcium, and chloride ions separately.

(76) $$R_{\mathrm{Na}}^{\mathrm{EM}}=-\nabla \cdot J_{\mathrm{Na}}^{\mathrm{EM}}=\left(\theta_{s}D_{\mathrm{Na}}C_{\mathrm{Na}}\right)\Delta \Psi +\nabla \left(\theta_{s}D_{\mathrm{Na}}C_{\mathrm{Na}}\right)\cdot \nabla \Psi ,$$

with similar expressions for $J_{\mathrm{Ca}}^{\mathrm{EM}}$, $R_{\mathrm{Ca}}^{\mathrm{EM}}$, $J_{\mathrm{Cl}}^{\mathrm{EM}}$, and $R_{\mathrm{Cl}}^{\mathrm{EM}}$.

**Figure 8:**
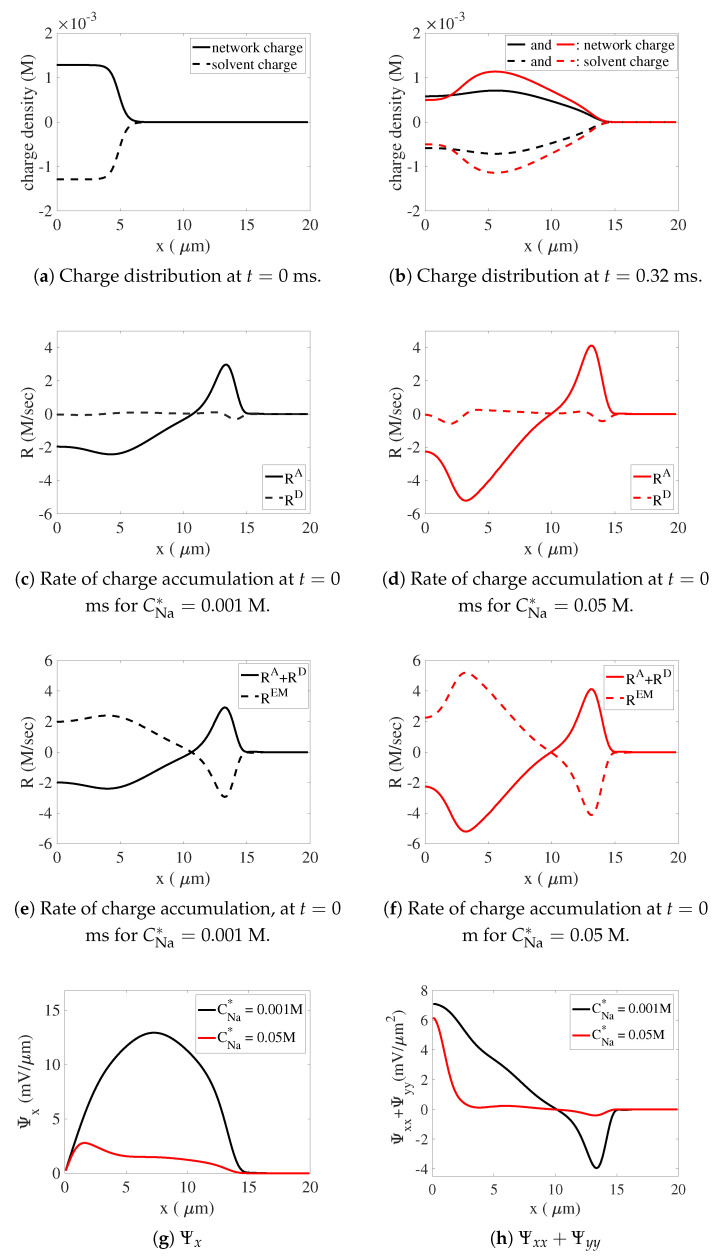
Distribution of charge-related variables along the positive x-axis. (**a**) Initial charge distributions on the network and solvent. All other plots are at t=0.32 ms. (**b**) Charge distributions on the network and solvent $C_{\mathrm{Na}}^{*}=0.001$ M and $C_{\mathrm{Na}}^{*}=0.05$ M. (**c**,**d**) The rates of charge accumulation due to advective and diffusive transport of charged species. (**e**,**f**) The rates of charge accumulation due to the combination of advection and diffusion and to electromigration. The variation of (**g**) $\Psi_{x}$ and (**h**) $\Psi_{xx}+\Psi_{yy}$ along the positive x-axis.

The distributions of charge density on the network and the solvent at t=0 ms and t=0.32 ms are plotted in Figure 8a,b for the low- and high-sodium cases. As the gel swells, the charge distributions for both simulations are much extended in space relative to their initial profiles. For the reasons discussed above, there is greater charge polarity between the network and solvent in the high-sodium case, that is, there is a larger positive charge on the network and a larger magnitude negative charge on the solvent in that case. The distributions of $R^{A}$ and $R^{D}$ from the two simulations are plotted in Figure 8c,d. We see that for both sodium bath concentrations, the advective transport makes a much larger magnitude contribution to the rate of local charge accumulation than does diffusion. Since the positively charged network is moving out, i.e., in the positive x-direction, while the negatively charged solvent is moving in the opposite direction toward the center of the gel, their motion, if not compensated for, would contribute to an accumulation of negative charges near the center of the gel and an accumulation of positive charges close to the gel’s edge. Further, because the positive charge on the network (and equal negative charge on the solvent) is larger for the high-sodium case, relative motion of the network and solvent would lead to greater charge accumulations in that case. The relative motions of network and solvent would lead to violation of the electroneutrality condition.

To maintain the electroneutrality condition, the local charge accumulations from advection and diffusion must be counterbalanced by the electromigration of the dissolved ions. In Figure 8e,f, the dashed curves show the contribution to charge accumulation due to the combination of advective and diffusive transport and we see the greater shifts of charge for the high-sodium case than for the low-sodium case. The solid curves show the local charge accumulation due to the electromigration flux. These plots show that for both simulations, the sum of the contributions to the charge accumulation is very close to zero, indicating that the electroneutrality condition is maintained by the electric fields induced in each simulation. For the high-sodium simulation, a more rapid charge accumulation from electromigration fluxes is required to counterbalance the more than two-fold larger value of $R^{A}+R^{D}$.

In Figure 8g,h, we plot $\Psi_{x}$ and $\Delta \Psi \equiv \Psi_{xx}+\Psi_{yy}$ at t=0.32 ms for the low- and high-sodium cases. We see that for both cases, $\Psi_{x}>0$ along the positive x-axis, and that the electric field is much weaker for the high-sodium simulation. In fact, the peak value of $\Psi_{x}$ in the low-sodium case is more than 4 times that in the high-sodium case. The values of $\Psi_{x}$ shown correspond to between ≈20 mV and ≈100 mV potential differences between the center of the gel and the bath, consistent with values reported in the literature [3]. We see that ΔΨ can have either sign. It is positive between x=0 and x≈10, and negative between there and the edge of the gel at x≈14. For high sodium, ΔΨ is large and positive only very close to the center of the gel, while for low sodium, ΔΨ has large positive values over a much larger x-interval. For x<10, ΔΨ is larger for low sodium than for high sodium. For 10<x<14, the magnitude of ΔΨ is ≈10-fold larger for low sodium than for high sodium.

To understand how the weak electric field in the high-sodium case can propel more than 2-fold greater electromigration while also giving rise to a much smaller magnitude electrical force, we consider the rate of change in local charge resulting from the electromigration of sodium ions as given by Equation (76), and which along the x-axis can be written

(77) $$R_{\mathrm{Na}}^{\mathrm{EM}}=\left(\theta_{s}D_{\mathrm{Na}}C_{\mathrm{Na}}\right)\Delta \Psi +{\left(\theta_{s}D_{\mathrm{Na}}C_{\mathrm{Na}}\right)}_{x}\Psi_{x}$$

because $\Psi_{y}=0$ there by symmetry. We also consider the contribution to the electric force $f_{s}^{E}$ on the solvent from the dissolved sodium ions,

(78) $$f_{s,\mathrm{Na}}^{E}=-\frac{k_{B}T}{\nu_{s}s^{\mathrm{tot}}}\theta_{s}C_{\mathrm{Na}}\Psi_{x}.$$

The two terms making up $R_{\mathrm{Na}}^{\mathrm{EM}}$ in Equation (77) and the expression for $f_{s,\mathrm{Na}}^{E}$ in Equation (78) all involve products of the sodium concentration $C_{\mathrm{Na}}$ or its derivative ${\left(C_{\mathrm{Na}}\right)}_{x}$ with the electric field $\nabla \Psi =(\Psi_{x},0)$ or its divergence ΔΨ=∇·∇Ψ, but the products in the three terms are distinct, and hence can have very different sizes depending on both the levels and variations in ion concentrations and on both the strength of the electric field and its divergence.

**Figure 9:**
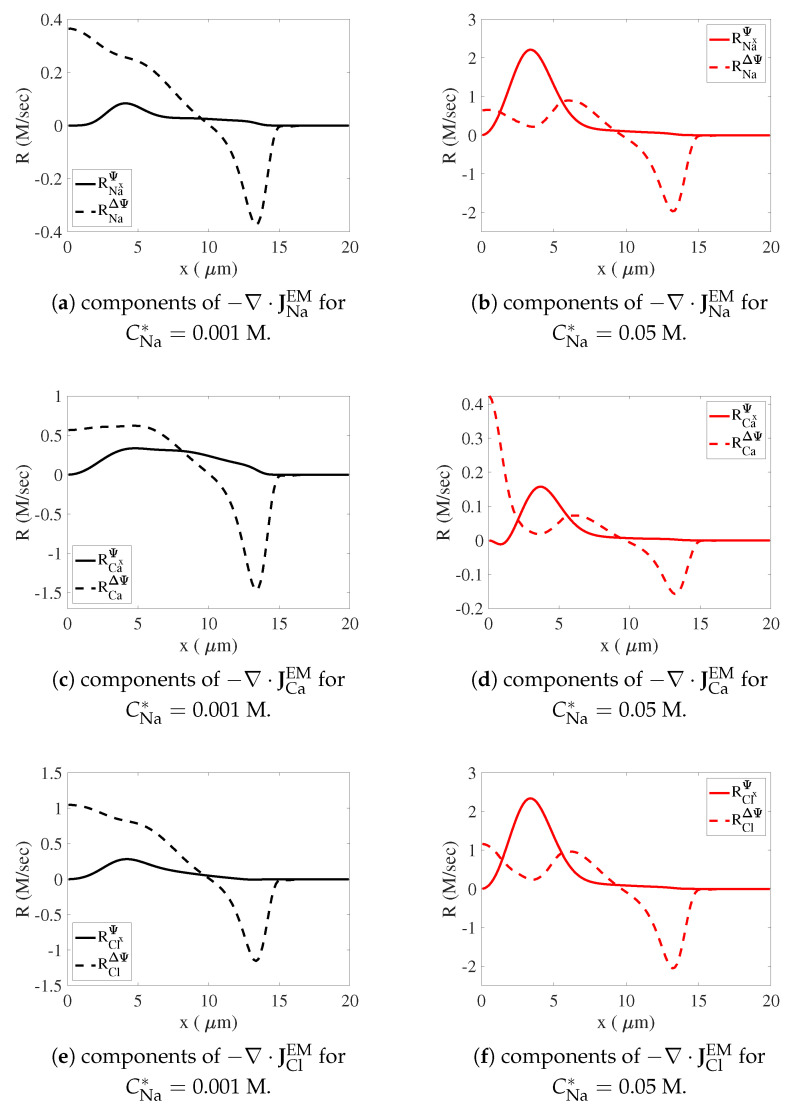
Rates of charge accumulation due to electromigration of various ions at t=0.32 ms. All solid curves represent the contribution from terms containing $\Psi_{x}$. All dashed curves represent the contribution from terms containing ΔΨ.

In Figure 9, we plot the two terms $(\theta_{s}D_{\mathrm{Na}}C\mathrm{Na})\Delta \Psi$ and ${\left(\theta_{s}D_{\mathrm{Na}}C\mathrm{Na}\right)}_{x}\Psi_{x}$ in Equation (77) for $R_{\mathrm{Na}}^{\mathrm{EM}}$ and the corresponding terms in the analogous expressions for $R_{\mathrm{Ca}}^{\mathrm{EM}}$ and $R_{\mathrm{Cl}}^{\mathrm{EM}}$ at t=0.32 ms for both the low- and high-sodium simulations. In Figure 9a,c,e, we see that for low sodium, the overall rate of accumulation of positive charge for x<10 seen in Figure 8e is mostly attributable to the $(\theta_{s}DC)\Delta \Psi$ terms for sodium, calcium, and chloride with much smaller contributions from ${\left(\theta_{s}D_{\mathrm{Na}}C_{\mathrm{Na}}\right)}_{x}\Psi_{x}$ and the corresponding terms for calcium and chloride. For high sodium, Figure 9b,d,f show that the overall accumulation of positive charge for x<10 seen in Figure 8f is almost entirely attributable to the ${\left(\theta_{s}D_{\mathrm{Na}}C_{\mathrm{Na}}\right)}_{x}\Psi_{x}$ and ${\left(\theta_{s}D_{\mathrm{Cl}}C_{\mathrm{Cl}}\right)}_{x}\Psi_{x}$ terms for the subregion 2<x<6, where the gradients in the sodium and chloride concentrations are so large that they compensate for a weak electric field. The term $(\theta_{s}D_{\mathrm{Na}}C_{\mathrm{Na}})\Delta \Psi$ and the corresponding terms for calcium and chloride contribute most to the rate of charge accumulation for x<2 where ΔΨ is large and in 6<x<10 where the sodium and chloride concentrations are high.

At the edge of the gel, 10<x<14, the overall rates of negative charge accumulation, shown in Figure 8e,f for both low and high sodium are mostly due to the $(\theta_{s}D_{\mathrm{Na}}C_{\mathrm{Na}})\Delta \Psi$, $(\theta_{s}D_{\mathrm{Ca}}C_{\mathrm{Ca}})\Delta \Psi$, and $(\theta_{s}D_{\mathrm{Cl}}C_{\mathrm{Cl}})\Delta \Psi$ terms, with calcium and chloride being more important for low sodium, and sodium and chloride being more important for high sodium. The small contributions in this region from the ${\left(\theta_{s}DC\right)}_{x}\Psi_{x}$ terms actually contribute to positive charge accumulation. These latter terms are small in this region for both high and low sodium because $\Psi_{x}$ is small there for high sodium and ${\left(\theta_{s}C\right)}_{x}$ is small there for all of the dissolved ions for low sodium.

In terms of the relative size of the dominant contributors for the low- and high-sodium cases, it is interesting to look at the $(\theta_{s}D_{\mathrm{Cl}}C_{\mathrm{Cl}})\Delta \Psi$ terms in the region 10<x<14 for both cases. For high sodium this term is approximately 5 times its value in the low-sodium case, even though ΔΨ is an order of magnitude smaller for high sodium, as observed earlier. This is possible because the small magnitude ΔΨ is multiplied by the ≈50-fold higher value of $\theta_{s}C_{\mathrm{Cl}}$ in 10<x<14 for high sodium.

## 5. Discussion

In this work, we have derived a model of mucus-like polyelectrolyte gels. In particular, our model accounts for the differing chemical affinities of the network polymers for various mono- and divalent ions, and the way in which this chemistry impacts the various forces that govern the dynamics of swelling of the gel. Simulation of the model was carried out using techniques that we had previously developed for a similar polyelectrolyte gel model [20]. In our numerical experiments, we placed a sample of dense, highly cross-linked gel containing a high concentration of calcium into a bath containing a high concentration of monovalent sodium and a much lower concentration of divalent calcium. We quantified the rate of swelling as a function of the bath sodium concentration. It was shown that the rate of swelling is (at early times) an increasing function of the sodium concentration.

The dependence of swelling speed on bath sodium concentration can be understood as a competition between several forces. Namely, as the bath concentration of sodium is increased, sodium more readily displaces calcium ions associated with the network, breaking cross-links and altering the short-range interaction potential, producing a larger force density that drives swelling. However, relative motion of the network and solvent (which both carry net charge) would lead to charge accumulation, which is prohibited by electroneutrality. Thus, an electric field is induced that rearranges charged species (both dissolved and associated to the network) in a way that maintains local charge balance. This electric field produces a force density on the network (and solvent) phase that resists swelling. Counterintuitively, in the case of high bath sodium, a relatively weak electric field can drive a relatively strong electromigration of dissolved ions to keep the gel electrically neutral. This in turn reduces the magnitude of the electric force density resisting relative motion of the phases, thus allowing rapid swelling to occur. Our simulation results show that the induced electric field is strongly affected by the swelling dynamics, the chemical binding kinetics and the distribution of charged species.

We note here that in this investigation, we have focused on a somewhat restricted subset of possible chemical parameters. In particular, we have assumed that the dissociation constant of network–sodium binding is always greater than the dissociation constant of network–calcium binding. Thus, binding with calcium is chemically preferred. There is some indication that this is the case in mucus networks [4]. However, attempts to measure the affinities of sodium and calcium binding with mucus have indicated that mucus networks may exhibit multiple distinct sites to which cations can associate, and that they may have distinct chemical characteristics [2]. Further complicating the issue, it appears that these parameters may be pH-dependent [31]. As such, this work may be something of an approximation to the chemistry-dependent swelling of mucus. However, the modeling framework is by no means restricted to calcium preferred chemistry, and may be used in future investigations of swelling behavior in chemically distinct parameter regimes.

Finally, while the work presented here was meant to understand the swelling behavior of a mucus-like gel, we note that neither the modeling framework, nor the simulation techniques we have employed are restricted to mucus dynamics. Indeed the modeling framework is extremely general, and may be employed to study a vast array of gels by altering, for example, the ionic species included, the binding/unbinding kinetic constants, the rheology of the network phase, or the short-range interaction energies of the solvent and network. Thus, this framework provides a very general tool with which to study many dynamic swelling and deswelling phenomena of polyelectrolyte gels.

The details of the numerical algorithm are discussed in [20]. Please contact jdu@fit.edu to inquire about obtaining a copy of the code used for the simulations in this paper.

## Figures and Tables

**Table 1:** Simulation Parameters. $\epsilon_{\mathrm{us}}$, $\epsilon_{\mathrm{uu}}$, $\epsilon_{\mathrm{ss}}$, $\epsilon_{xx}$, and $\epsilon_{\mathrm{pp}}$ are the interaction energies nondimensionalized by $k_{B}T$.

Parameter	Value
network viscosity $\eta_{n}$	100 Poise [27]
solvent viscosity $\eta_{s}$	0.01 Poise
diffusion coefficient $D_{j}$	$2.5\times 10^{-5}\;{\mathrm{cm}}^{2}/s$ [28]
drag coefficient ξ/ν	$2.5\times 10^{9}\;g/\left({\mathrm{cm}}^{3}\cdot s\right)$
$\epsilon_{\mathrm{us}}-\epsilon_{\mathrm{uu}}$	−18.0
$\epsilon_{\mathrm{us}}-\epsilon_{\mathrm{ss}}$	10.0
$\epsilon_{xx}-\epsilon_{\mathrm{uu}}$	−0.5
$\epsilon_{\mathrm{pp}}-\epsilon_{\mathrm{uu}}$	0
number of monomers in a chain *N*	6
network charge density $m^{\mathrm{tot}}$	0.1Molar
size of monomer $\nu_{n}$	1.661 × 10${}^{-8}$ ${\mu m}^{3}$
coordination number *z*	6

**Table 2:** Initial values of model variables in the gel and bath.

Variable	Gel	Bath
$\theta_{n}$	0.5	0
$C_{\mathrm{Na}}$	$3.9912\times 10^{-4}\;M$	variable ($C_{\mathrm{Na}}^{*}$)
$C_{\mathrm{Ca}}$	$1.1414\times 10^{-4}\;M$	$1.0\times 10^{-3}\;M$
$C_{\mathrm{Cl}}$	$3.2\times 10^{-3}\;M$	variable
$B_{\mathrm{MNa}}$	$8.0\times 10^{-4}\;M$	0M
$B_{\mathrm{MCa}}$	$2.2891\times 10^{-3}\;M$	0M
$B_{M_{2}\mathrm{Ca}}$	$2.2954\times 10^{-2}\;M$	0M
